# Ferritin-armed extracellular vesicles with enhanced BBB penetration and tumor-targeting ability for synergistic therapy against glioblastoma

**DOI:** 10.1186/s12951-025-03646-x

**Published:** 2025-08-18

**Authors:** Guihong Lu, Peiling Zhuang, Feng Li, Fan Zhang, Xiaoyan Li, Weixiu Wang, Hui Tan

**Affiliations:** 1 Institute of Pediatrics, Shenzhen Children’s Hospital, Shenzhen, 518038 P. R. China; 2 Department of Neurosurgery, Health Science Center, Shenzhen Second People’s Hospital, The First Affiliated Hospital of Shenzhen University, Shenzhen, 518035 P. R. China; 3 State Key Laboratory of Biopharmaceutical Preparation and Delivery, Institute of Process Engineering, Chinese Academy of Sciences, Beijing, 100190 P. R. China

**Keywords:** BBB penetration, Dual-targeting, LA metabolic therapy, Multimodal therapy, Glioblastoma

## Abstract

Glioblastoma multiforme (GBM) is an aggressive brain cancer with a high mortality rate and limited treatment options. Metabolism-based synergistic therapy holds promise for GBM treatment, however, its efficacy is significantly impeded by poor blood-brain barrier (BBB) penetration, inadequate targeting of GBM cells, and systemic drug-related side effects. To address these challenges, we herein developed a dual-targeting nanoplatform, EVs@siMCT4-HFn@AuMn, by arming siMCT4-loaded M1-type microglia-derived extracellular vesicles (EVs) with ultrasmall nano-Au/MnO_2_-loaded H-ferritin (HFn). This nanoplatform enhances tumor accumulation through cooperative BBB penetration and the GBM-targeting properties of EVs and HFn. Within the GBM microenvironment, siMCT4 silences MCT4 expression, inhibitits lactate (LA) efflux, increases the intracellular LA levels to induce glioma cell apoptosis via LA metabolic therapy, and reduces extracellular LA to achieve M2-to-M1 polarization of tumor-associated macrophages for immunomodulation of the tumor microenvironment. Concurrently, the delivered ultrasmall nano-Au consumes glucose for starvation therapy and facilitates H_2_O_2_ production, which is utilized by the co-delivered ultrasmall nano-MnO_2_ to generate cytotoxic hydroxyl radicals (•OH), further enhancing tumor cell eradication. This synergistic approach effectively suppresses tumor growth in a glioma xenograft model with negligible side effects, highlighting the potential of EVs@siMCT4-HFn@AuMn as a flexible and powerful platform for metabolism-based multimodal GBM therapies.

**Graphical abstract:**

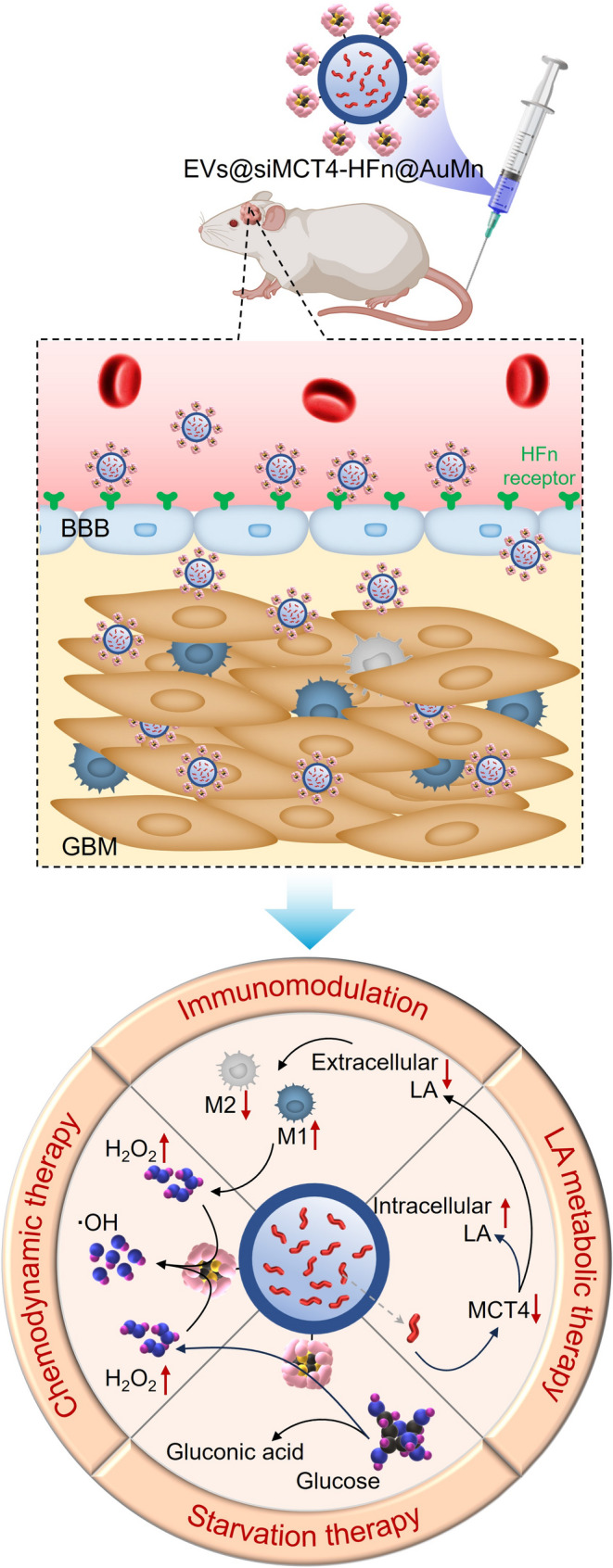

**Supplementary Information:**

The online version contains supplementary material available at 10.1186/s12951-025-03646-x.

## Introduction

Glioblastoma multiforme (GBM) is an aggressive primary intracranial tumor characterized by high morbidity and mortality. Despite advances in neurosurgical techniques, radiotherapy, and chemotherapy, no effective treatments are capable of eradicating this disease [1–3]. The specific location of the tumor within the brain, coupled with its diffuse infiltrative nature, makes complete surgical resection unattainable. Postoperative therapies provide some benefit; however, their efficacy is often limited by drug resistance and severe systemic toxicity [4, 5]. As a tumor with high glycolytic activity, GBM accumulates large quantities of lactate (LA) in the tumor microenvironment (TME), contributing to an acidic and immunosuppressive milieu [6, 7]. This accumulation of LA is strongly associated with tumor invasion, metastasis, and therapeutic resistance, including diminished efficacy of chemotherapy and immunotherapy [8–11]. Consequently, regulating LA metabolism has emerged as an attractive therapeutic strategy for GBM.

Monocarboxylate transporter 4 (MCT4) plays a pivotal role in exporting LA into the TME, exacerbating its acidity and immunosuppressive characteristics [12, 13]. Silencing MCT4 with small interfering RNA (siRNA) has the potential to disrupt LA metabolism, induce tumor cell apoptosis, and enhance the efficacy of chemoimmunotherapy [14–16]. However, the clinical application of naked siRNA is severely hindered by its poor stability, limited penetration across the blood-brain barrier (BBB), and lack of glioma-targeting specificity [17–19]. Additionally, the use of single-modality LA metabolic therapy is unlikely to achieve complete tumor eradication due to the highly aggressive and infiltrative nature of GBM [20, 21]. Therefore, there is an urgent need for innovative delivery systems that enhance siRNA targeting and therapeutic efficiency.

Recent advances in drug delivery have focused on developing nanoplatforms capable of actively crossing the BBB and selectively targeting glioma cells [22–24]. Although some chemically synthesized delivery systems have shown promise, their clinical translation is limited by complex synthesis procedures and potential long-term toxicity. In contrast, biomimetic vesicles, such as extracellular vesicles and protein-based nanoparticles (NPs), offer superior biocompatibility and safety [25–27]. Among these, M1-type microglia/macrophage-derived extracellular vesicles (M1EVs) and H-ferritin (HFn) have demonstrated notable potential in cancer therapy, including GBM [21, 28–30]. As endogenous components, M1EVs and HFn offer several advantages, including high biocompatibility, reduced immunogenicity, prolonged circulation times, and intrinsic BBB penetration and tumor-targeting abilities. Combining M1EVs and HFn into a dual-targeting system may further improve drug delivery efficiency, a concept that warrants exploration.

**Scheme 1:**
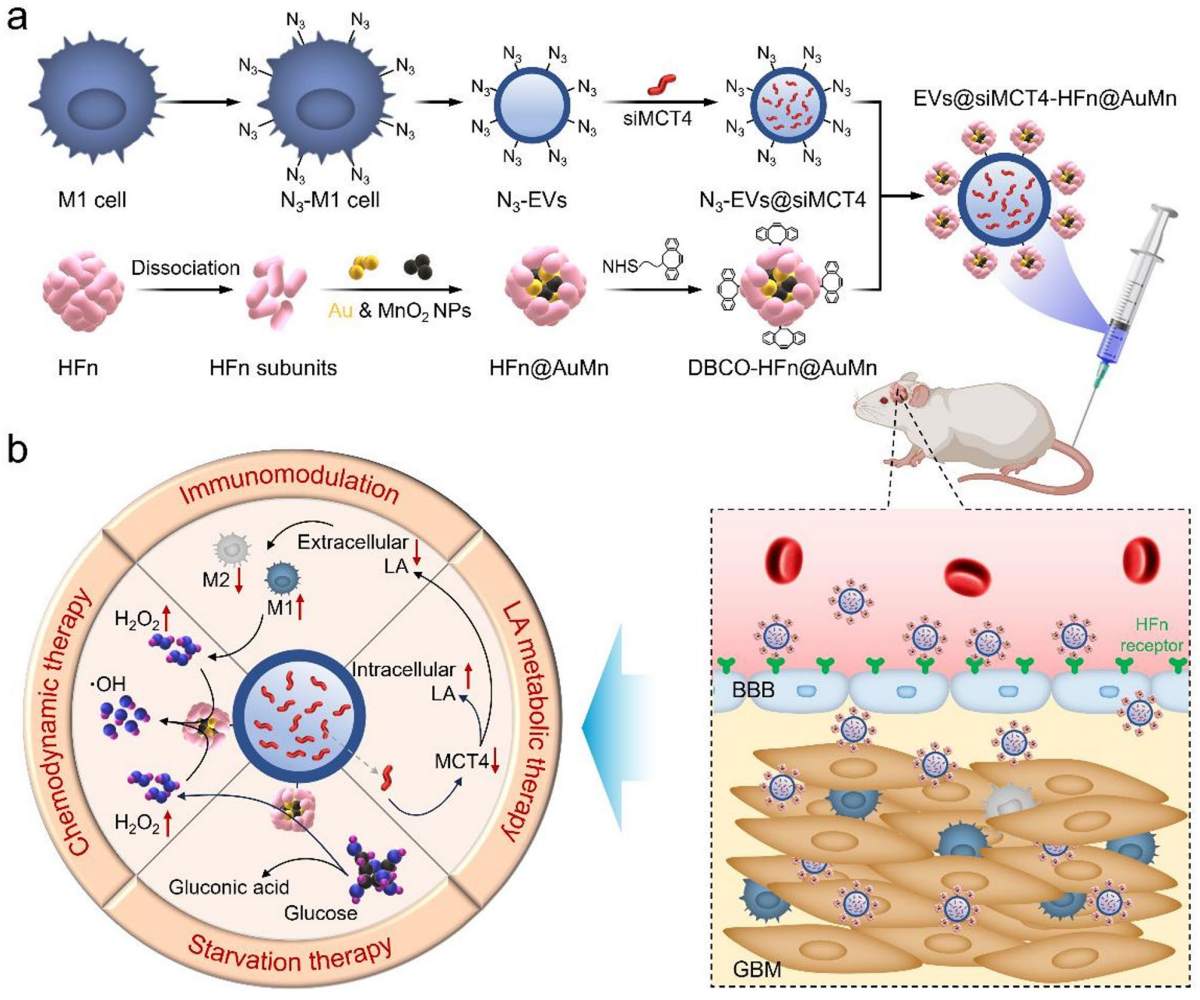
Schematic illustration of EV@siMCT4-HFn@AuMn construction for GBM inhibition. (**a**) Schematic illustration of the construction of EV@siMCT4-HFn@AuMn. (**b**) Targeted accumulation of EV@siMCT4-HFn@AuMn in GBM and subsequent synergistic therapy upon the combination of LA metabolic therapy, starvation therapy (ST), chemodynamic therapy (CDT), and immunomodulation

Here, we present the development and proof-of-concept demonstration of a dual-targeting nanoplatform, EVs@siMCT4-HFn@AuMn, designed to cross the BBB and selectively target GBM, where it functions as a versatile treatment platform for combined LA metabolic therapy, starvation therapy (ST), chemodynamic therapy (CDT), and immunomodulation (Scheme 1). Briefly, we armed azide-modified M1EVs (N_3_-EVs) with dibenzylcyclooctyne-modified HFn (DBCO-HFn) via click chemical to generate the EVs-HFn delivery system. After confirming the dual-targeting abilities, the EVs-HFn system was further loaded with siMCT4, as well as ultrasmall nano-Au/MnO_2_ (~ 3 nm), to create EVs@siMCT4-HFn@AuMn for synergistic therapy. The nanoplatform combines LA metabolic therapy and immunomodulation mediated by siMCT4 with glucose depletion induced by ultrasmall nano-Au for ST and hydroxyl radical (•OH) generation catalyzed by ultrasmall nano-MnO_2_ for CDT. This synergistic approach significantly inhibits GBM progression while maintaining excellent biosafety, highlighting the potential of EVs@siMCT4-HFn@AuMn as a multimodal therapeutic system for treating GBM. 

## Results and discussion

### Construction,** c**haracterization and BBB penetration ability of EV-HFn

**Fig. 1:**
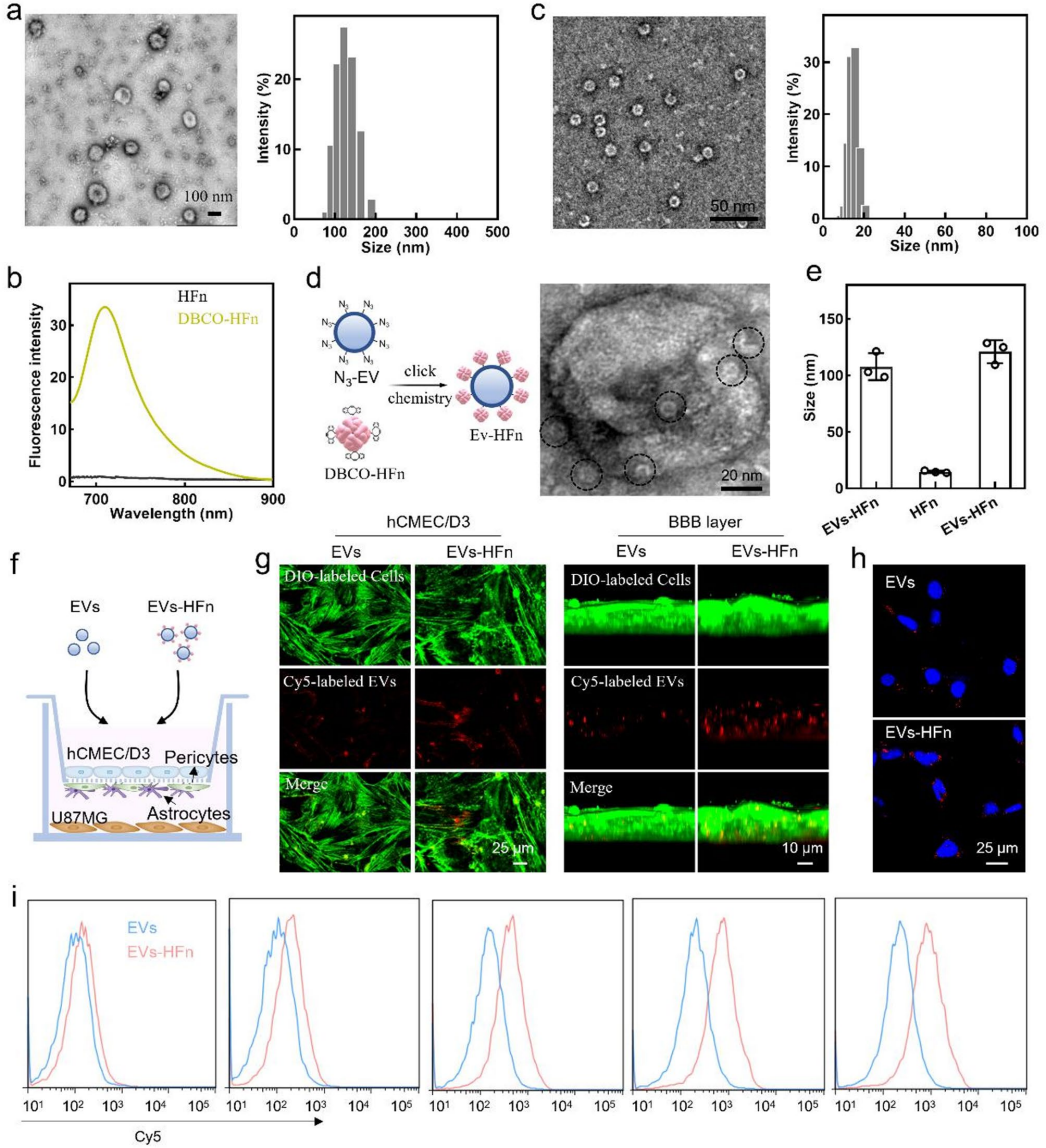
Construction of EVs-HFn and evaluation of their glioma cell targeting ability in vitro. (**a**) Representative TEM image (left) and hydrodynamic diameter distribution (right) of N_3_-EVs. (**b**) Fluorescence spectra of the HFn and DBCO-HFn samples after incubation with N_3_-Cy5.5. (c) Representative TEM image (left) and hydrodynamic diameter distribution (right) of DBCO-HFn. (**d**) Schematic illustration of the preparation (left) and representative TEM (right) of EVs-HFn. (**e**) Hydrodynamic diameters of N_3_-EVs, DBCO-HFn, and EVs-HFn. (**f**) Schematic illustration of the in vitro BBB model. (**g**) Representative CLSM images of the hCMEC/D3 monolayer showing the penetration of EVs or EVs-HFn. Red: Cy5-labeled EVs; Green: phalloidine-labeled hCMEC/D3 cells or BBB layer. (**h**) Representative CLSM images of U87MG cells showing the uptake of EVs or EVs-HFn. Red: Cy5-labeled EVs; Blue: Hoechst 33,342-labeled nuclei. (**i**) Flow cytometry histograms showed the Cy5 fluorescence signal in U87MG cells after incubation with Cy5-labeled EVs or EVs-HFn for the indicated periods. Data in e are presented as the mean ± SD (*n* = 3)

HFn was stably anchored onto EVs via click reaction to generate EV-HFn. To achieve this goal, we first modified EVs and HFn with azide (N_3_) and dibenzylcyclooctyne (DBCO) groups, respectively. Specifically, N_3_-modified EVs (N_3_-EVs) were isolated from the culture supernatant of N_3_-modified M1-type HMC3 cells using ultracentrifugation, as described in our previously published metabolic incorporation method [31, 32]. Transmission electron microscopy (TEM) revealed that N_3_-EVs exhibited the typical cup-like morphology, with an average diameter of approximately 120 nm (Fig. 1a). Concurrently, we prepared DBCO-modified HFn (DBCO-HFn) by reaching the amino (NH_2_) groups on HFn and the N-hydroxysuccinimide (NHS) groups in NHS-PEG_5_-DBCO molecules. The successful modification of DBCO was confirmed by the appearance of a fluorescence peak in the DBCO-HFn sample after coincubation with N_3_-Cy5.5 (Fig. 1b). Moreover, DBCO modification did not affect the morphology and particle size of HFn (Fig. 1c and Fig S1a). Subsequently, N_3_-EVs were conjugated with DBCO-HFn through a click chemistry reaction to generate EVs-HFn. The TEM image, along with the slight changes in size and zeta potential between EVs-HFn and EVs, confirmed the successful conjugation of HFn onto the EVs (Fig. 1d-e and Fig. S1b). The loading efficiency of HFn on EVs-HFn was determined to be 24.1%.

Given the overexpression of transferrin receptor protein 1 (TFR1, the natural receptor of HFn) in both BBB endothelial cells and glioma cells, we reasoned that arming EVs with HFn would enhance their BBB penetration and glioma targeting capabilities. To explore this, we assessed the BBB penetration capacity of EV-HFn using a classic in vitro BBB model [6]. As shown in Fig. 1f, the transwell™ co-culture system containing a monolayer (BBB layer) of human brain endothelial cell hCMEC/D3 cells and a mixed layer of brain pericytes and astrocytes in the upper chamber, and U87MG human glioma cells in the bottom chamber. After the hCMEC/D3 cells formed a monolayer, Cy5-labeled EVs or EVs-HFn were added into the upper chamber. The uptake of EVs or EVs-HFn by hCMEC/D3 and U87MG cells was analyzed using confocal laser scanning microscopy (CLSM). Compared with those of EVs, markedly stronger fluorescence signals were observed in the CLSM images of hCMEC/D3, BBB layer, and U87MG cells after exposure to EVs-HFn (Fig. 1g-h), which should be attributed to the HFn receptor-mediated transport (Fig. S2) [29]. Additionally, flow cytometry analysis at different time points revealed that the uptake of EVs-HFn by U87MG cells was consistently higher than that of EVs, and this difference in endocytosis increases over time (Fig. 1i and Fig. S3). At 1 h, the intracellular EVs-HFn were 1.4-fold higher than that of EVs, and this value increased to 3.9-fold at 24 h. These results suggested that arming EVs with HFn enhanced both their BBB penetration and glioma cell targeting capabilities.

### In vivo BBB penetration and GBM-targeting performance evaluation

**Fig. 2:**
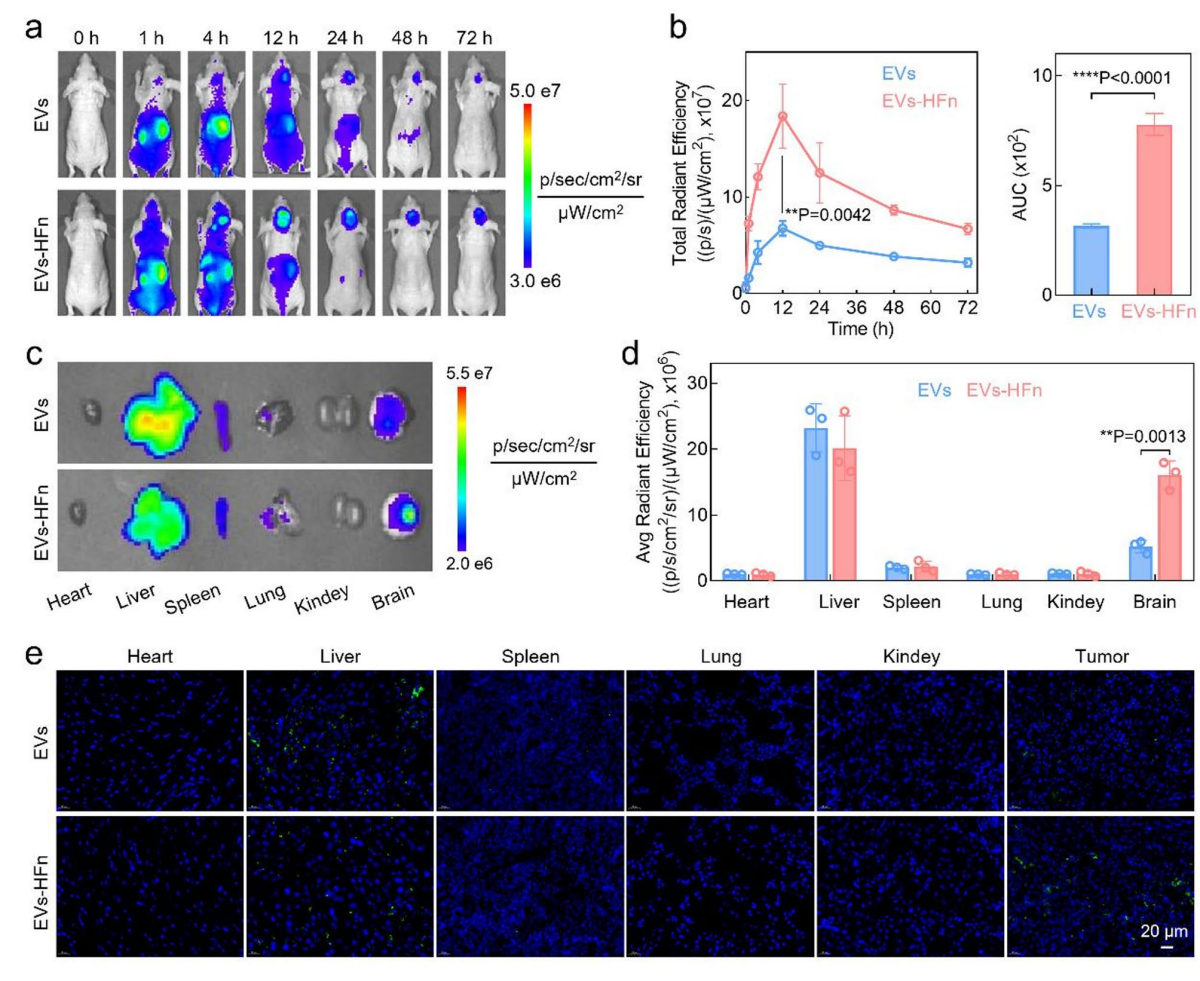
Evaluation of the glioma targeting ability of EVs-HFn in an orthotopic U87MG-luc glioma model. (**a**) Real-time fluorescence imaging of glioma-bearing mice after *i.v.* injection of DIR-labeled EVs or EVs-HFn. (**b**) Signal profiles of EVs or EVs-HFn in glioma tumors and the corresponding AUC calculations. (**c**) Fluorescence images of the main organs and brains excised from the indicated groups at 24 h after drug administration. (**d**) Corresponding quantitative analysis of fluorescence intensity in major organs and gliomas. (**e**) Representative fluorescence images of frozen major organ and glioma tumor sections from the indicated groups at 24 h after administration. Blue: Hoechst 33,342-labeled nuclei; green: DIO-labeled EVs. Data in b and d are presented as the mean ± SD (*n* = 3). P values are calculated using two-tailed unpaired Student’s *t*-test

To assess the potential BBB penetration and GBM-targeting ability of EVs-HFn in vivo, we intravenously injected DIR-labeled EVs or EVs-HFn into U87MG-Luc glioma-bearing Balb/c nude mice. The biodistribution of the treatments was monitored using an IVIS imaging system at designated time points. As shown in Fig. 2a-b, although both EVs and EVs-HFn targeted the GBM, the fluorescence signals from the EVs-HFn group were substantially brighter at all imaging time points, with a 2.5-fold higher area under the curve (AUC) than those of the EVs group. This enhanced targeted accumulation of EVs-HFn was further confirmed by ex vivo imaging of dissected main organs (heart, liver, etc.) and tumor-bearing brains, where much stronger fluorescence was observed in the tumors of EVs-HFn-treated mice than of EVs-treated mice (Fig. 2c). Further quantitative analysis revealed that the accumulation of EVs-HFn in GBM-containing brains was 3.1 times higher than that of EVs (Fig. 2d). We also examined the corresponding frozen sections and found that EVs-HFn exhibited substantial accumulation in the glioma tissues, whereas minimal EVs signals were detected in the glioma region (Fig. 2e). These results underscore the good BBB penetration and GBM-targeting capabilities of EVs-HFn in vivo.

### Construction and characterization of EVs@siMCT4-HFn@AuMn

**Fig. 3:**
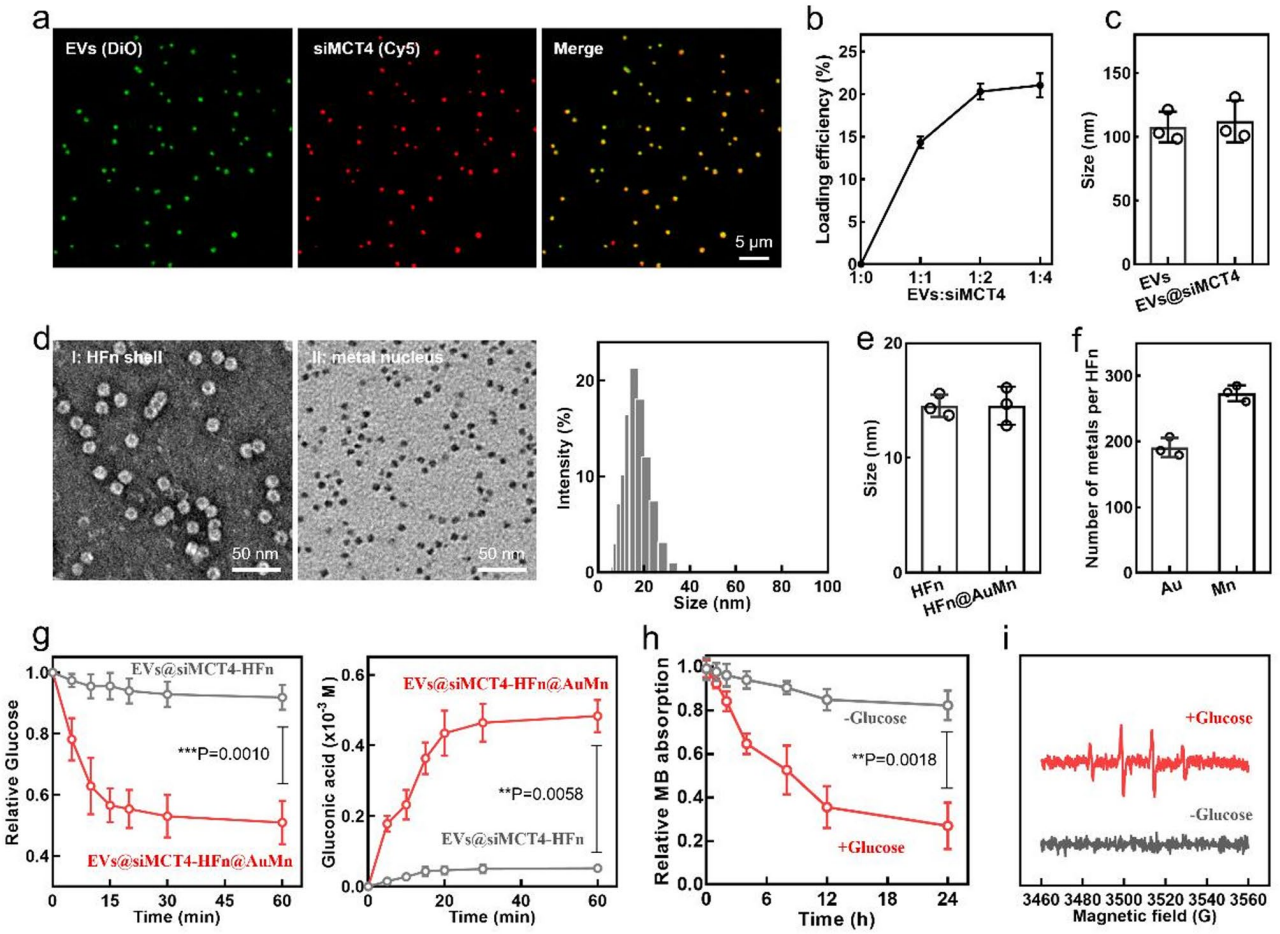
Construction and characterization of EVs@siMCT4-HFn@AuMn. (**a**) CLSM images of EVs@siMCT4. (**b**) Loading efficiencies of siMCT4 in EVs@siMCT4 at the indicated ratios of EVs to siMCT4 (w/w). (**c**) Hydrodynamic diameters of EVs and EVs@siMCT4. (**d**) Representative TEM images of the HFn shell and metal nucleus (left), and the hydrodynamic diameter distribution (right) of HFn@MnAu. HFn@MnAu was imaged directly (I) or negatively stained with 2% phosphotungstic acid solution (II). (**e**) Hydrodynamic diameters of HFn and HFn@MnAu. (**f**) Quantification of the Au and Mn contents in a single HFn@MnAu. (**g**) Relative glucose (left) and gluconic acid (right) concentration change curves of glucose solutions incubated with the indicated formulations. (**h**) Absorption intensity changes of MB in EVs@siMCT4-HFn@AuMn solutions with or without glucose. (i) ESR spectroscopy of EVs@siMCT4-HFn@AuMn solutions with or without glucose. Data in b, c, and e-h are presented as the mean ± SD (*n* = 3). P values are calculated using two-tailed unpaired Student’s *t*-test

After demonstrating the enhanced GBM-targeting performance of EVs-HFn, we sought to load antitumor drugs into EVs-HFn and assess their potential antitumor functionality. First, we loaded siMCT4 into N_3_-EVs to prepare siMCT4-loaded EVs (EVs@siMCT4) *via* electroporation. As shown in Fig. 3a, Cy5-labeled siMCT4 colocalized well with 3,3′-dioctadecyloxacarbocyanine perchlorate (DiO)-labeled N_3_-EVs, suggesting the successful loading of siMCT4. After optimization, the loading efficiency of siMCT4 was determined to be 20.3% (Fig. 3b and Fig. S4). Additionally, the loading of siMCT4 did not influence the particle size of N_3_-EVs (Fig. 3c). Next, we synthesized ultrasmall nano-Au and MnO_2_ according to previously established protocols [33, 34]. Both the Au and MnO_2_ NPs exhibited monodispersity with diameters of approximately 3 nm (Fig S5). We then embedded these ultrasmall nano-Au and MnO_2_ into HFn based on the disassembly and self-assembly capability of HFn to prepare Au/MnO_2_-loaded HFn (HFn@AuMn) [35]. The presence of ultrasmall NPs in the TEM images demonstrated the successful preparation of HFn@AuMn, with no change in the morphology or particle size of HFn (Fig. 3d-e). Further analysis using inductively coupled plasma (ICP) mass spectrometry revealed the presence of both Au and Mn in HFn@AuMn, indicating successful incorporation of these two types of ultrasmall NPs. The contents of Au and Mn were approximately 191 and 274 metals per HFn, respectively (Fig. 3f). Finally, we constructed EVs@siMCT4-HFn@AuMn through the aforementioned click chemistry reaction between EVs@siMCT4 and HFn@AuMn.

Having EVs@siMCT4-HFn@AuMn in hand, we subsequently evaluated their potential multiple antitumor functionalities. To assess the glucose consumption activity exhibited by ultrasmall nano-Au in EVs@siMCT4-HFn@AuMn, we incubated EVs@siMCT4-HFn (lacking ultrasmall nano-Au/MnO_2_) or EVs@siMCT4-HFn@AuMn with a glucose solution and monitored the glucose and gluconic acid concentrations at different time points. As shown in Fig. 3g, the EVs@siMCT4-HFn@AuMn group exhibited time-dependent efficient glucose consumption and gluconic acid production, whereas EVs@siMCT4-HFn did not show these activities, indicating that EVs@siMCT4-HFn@AuMn possessed the desired glucose consumption capabilities. We also investigated the ability of EVs@siMCT4-HFn@AuMn to convert H_2_O_2_ into hydroxyl radicals (•OH) *via* the chemodynamic effect induced by manganese (Mn) ions. We anticipated that ultrasmall nano-Au would catalyze the conversion of intracellular glucose, which generated H_2_O_2_ and facilitate the chemodynamic effect. To confirm this, 1 mM glucose was incubated with the indicated formulations and methylene blue (MB) was used as a probe to detect •OH production during the catalytic process. As expected, we observed a clear reduction in the MB absorption intensity over time when EVs@siMCT4-HFn@AuMn was incubated with glucose (Fig. 3h), indicating substantial •OH generation. This result was further verified by electron spin resonance (ESR) spectroscopy, where the appearance of characteristic peaks after the addition of glucose confirmed •OH generation (Fig. 3i). Furthermore, EVs@siMCT4-HFn@AuMn exhibited BBB penetration capability, with efficiency comparable to unmodified EVs-HFn (Fig. S6 and Fig. S7a-c). Notably, these nanoparticles exhibited robust glioma-specific accumulation while showing minimal distribution in adjacent normal brain tissue (Fig. S7d). Quantitative analysis also revealed that the concentrations of Au and Mn delivered to glioma tissue reached 6.88% and 7.49% ID/g, respectively, significantly higher than those in normal brain tissue (Fig. S7e). These results unequivocally demonstrate that EVs@siMCT4-HFn@AuMn retained its BBB penetration and GBM-targeting properties even after drug encapsulation.

### In vitro synergistic antitumor efficacy of EVs@siMCT4-HFn@AuMn

**Fig. 4:**
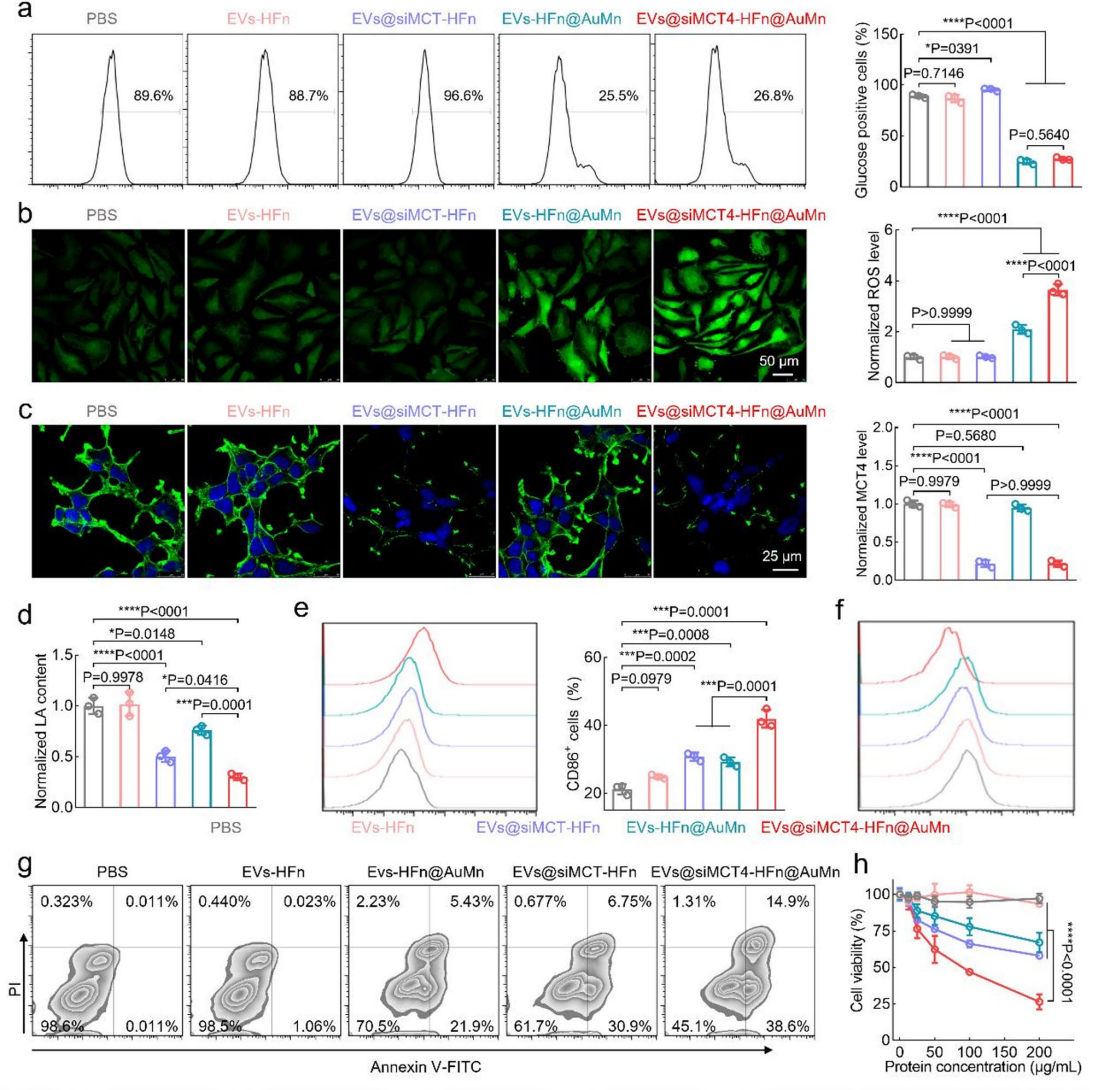
Evaluation of multiple therapeutic modalities of EVs@siMCT4-HFn@AuMn in vitro using U87MG glioma cells. (**a**) Flow cytometry and corresponding quantitative analysis of the intracellular glucose content in U87MG cells after incubation with PBS, EVs-HFn, EVs@siMCT4-HFn, EVs-HFn@MnAu, or EVs@siMCT4-HFn@AuMn. (**b**) CLSM images and corresponding quantitative analysis of ROS levels in U87MG cells after the indicated treatments. (**c**) CLSM images and corresponding quantitative analysis of MCT4 expression in U87MG cells after the indicated treatments. (**d**) Normalized extracellular LA contents after the indicated treatments. (**e**) Flow cytometry analysis of CD86 expression in macrophages and the corresponding quantification of the percentage of CD86-positive macrophages after the indicated treatments. (**f**) Flow cytometry analysis of CD206 expression in macrophages after the indicated treatments. (**g**) Flow cytometry analysis of the extent of U87MG cell apoptosis based on Annexin V/PI staining. (**h**) Cell viability of U87MG after the indicated treatments. Data in a-e, h are presented as the mean ± SD (*n* = 3). P values are calculated using one-way ANOVA

Next, we explored the in vitro antitumor activity of EVs@siMCT4-HFn@AuMn. In addition to EVs@siMCT4-HFn@AuMn, we also prepared EVs-HFn (lacking siMCT4 and ultrasmall nano-Au/MnO_2_), EVs-HFn@AuMn (lacking siMCT4), and EVs@siMCT4-HFn (lacking ultrasmall nano-Au/MnO_2_). We first evaluated the glucose consumption effect by measuring the intracellular glucose levels in U87MG cells incubated with the various formulations using a glucose uptake and transport probe, 2-deoxy-2-[(7-nitro-2,1,3-benzoxadiazol-4-yl) amino]-D-glucose (2-NBDG). As shown in Fig. 4a, no significant change in fluorescence intensity was observed in cells treated with EVs-HFn (lacking siMCT4 and ultrasmall nano-Au/MnO_2_), compared with those in the PBS group. However, in the groups containing ultrasmall nano-Au (EVs-HFn@AuMn and EVs@siMCT4-HFn@AuMn), the fluorescence intensity from 2-NBDG was significantly decreased, demonstrating glucose consumption. Focusing on CDT, we measured •OH production using 2′,7′-dichlorofluorescin diacetate (DCFH-DA) as a probe after the indicated treatment. In contrast to EVs-HFn and EVs@siMCT4-HFn-treated cells, cells treated with EVs-HFn@AuMn (lacking siMCT4) and EVs@siMCT4-HFn@AuMn presented markedly brighter fluorescence (Fig. 4b). Notably, the fluorescence intensity in the EVs@siMCT4-HFn@AuMn group was 1.7-fold higher than that in the EVs-HFn@AuMn group, primarily due to the additional generation of H_2_O_2_ during glucose consumption induced by ultrasmall nano-Au.

To evaluate the silencing effect of siMCT4 on MCT4 expression, we incubated U87MG cells with the indicated formulations and assessed MCT4 expression in cells using immunofluorescence. CLSM images revealed that treatment with EVs-HFn or EVs-HFn@AuMn had no significant effect on MCT4 expression, as their fluorescence intensity was strong and similar to that of the PBS group (Fig. 4c). However, when the cells were treated with EVs@siMCT4-HFn or EVs@siMCT4-HFn@AuMn, the fluorescence signal substantially decreased, demonstrating effective silencing of MCT4 expression by the siMCT4-loaded vesicles. MCT4 silencing led to a reduction in extracellular LA levels, as evidenced by lower LA levels in the coculture medium of EVs@siMCT4-HFn-treated cells compared to the PBS-treated group (Fig. 4d). More importantly, the EVs@siMCT4-HFn@AuMn group exhibited further reduced extracellular LA levels, combining the effects of siMCT4-induced inhibition of LA excretion and the suppression of LA production through ultrasmall nano-Au-mediated glucose consumption. Meanwhile, intracellular LA levels were higher in EVs@siMCT4-HFn and EVs@siMCT4-HFn@AuMn-treated U87MG cells than in the other groups (Fig. S8). These results demonstrate that EVs@siMCT4-HFn@AuMn effectively silenced MCT4 expression, inhibited LA excretion, and increased the level of intracellular LA, potentially inducing tumor cell apoptosis [14]. These findings underscore the potential of LA metabolic therapy in U87MG cells.

Given that extracellular LA can influence the polarization of the TAMs phenotype [14], we assessed the impact of LA on TAMs polarization using a Transwell™ coculture system with U87MG cells in the upper chamber and macrophages in the lower chamber. After the various formulations were added into the upper chamber and incubated for 24 h, we analyzed the expression of M1-type and M2-type macrophages using flow cytometry. We observed that treatment with EVs@siMCT4-HFn, EVs-HFn@AuMn, or EVs@siMCT4-HFn@AuMn increased the proportion of M1-type macrophages and decreased the proportion of M2-type macrophages (Fig. 4e-f and Fig. S9). Notably, the EVs@siMCT4-HFn@AuMn group presented the highest proportion of CD86-positive (M1-type) macrophages and the lowest proportion of CD206-positive (M2-type) macrophages, which corresponded to the lowest extracellular LA levels. These findings suggest that the decreased extracellular LA induced by EVs@siMCT4-HFn@AuMn promote the polarization of TAMs from the immunosuppressive M2-type to the immune-activated M1-type, potentially enabling their immunomodulatory effects.

The efficient glucose consumption, •OH generation, MCT4 silencing, and TAMs polarization abilities of EVs@siMCT4-HFn@AuMn inspired us to evaluate their synergistic effects in the context of ST, CDT, LA metabolic therapy, and immunomodulation against U87MG glioma cells in the Transwell™ coculture system. We found that EVs-HFn@AuMn and EVs@siMCT4-HFn treatments mildly induced cell cytotoxicity, with 29.6% and 38.3% of the cells undergoing apoptosis after 24 h of incubation, respectively (Fig. 4g). However, owing to the synergistic effect, the percentage of apoptotic cells increased to 54.8% in the EVs@siMCT4-HFn@AuMn group. This enhanced antitumor activity was further confirmed by CCK8 assays (Fig. 4h), where EVs@siMCT4-HFn@AuMn exhibited significantly enhanced cytotoxicity with an IC50 of 85.8 µg/mL, representing a 2.5-fold and 4.3-fold increase in potency compared to EVs@siMCT4-HFn (212.5 µg/mL) and EVs-HFn@MnAu (364.7 µg/mL), respectively.

### Therapeutic effects of EVs@siMCT4-HFn@AuMn on CDX model

**Fig. 5:**
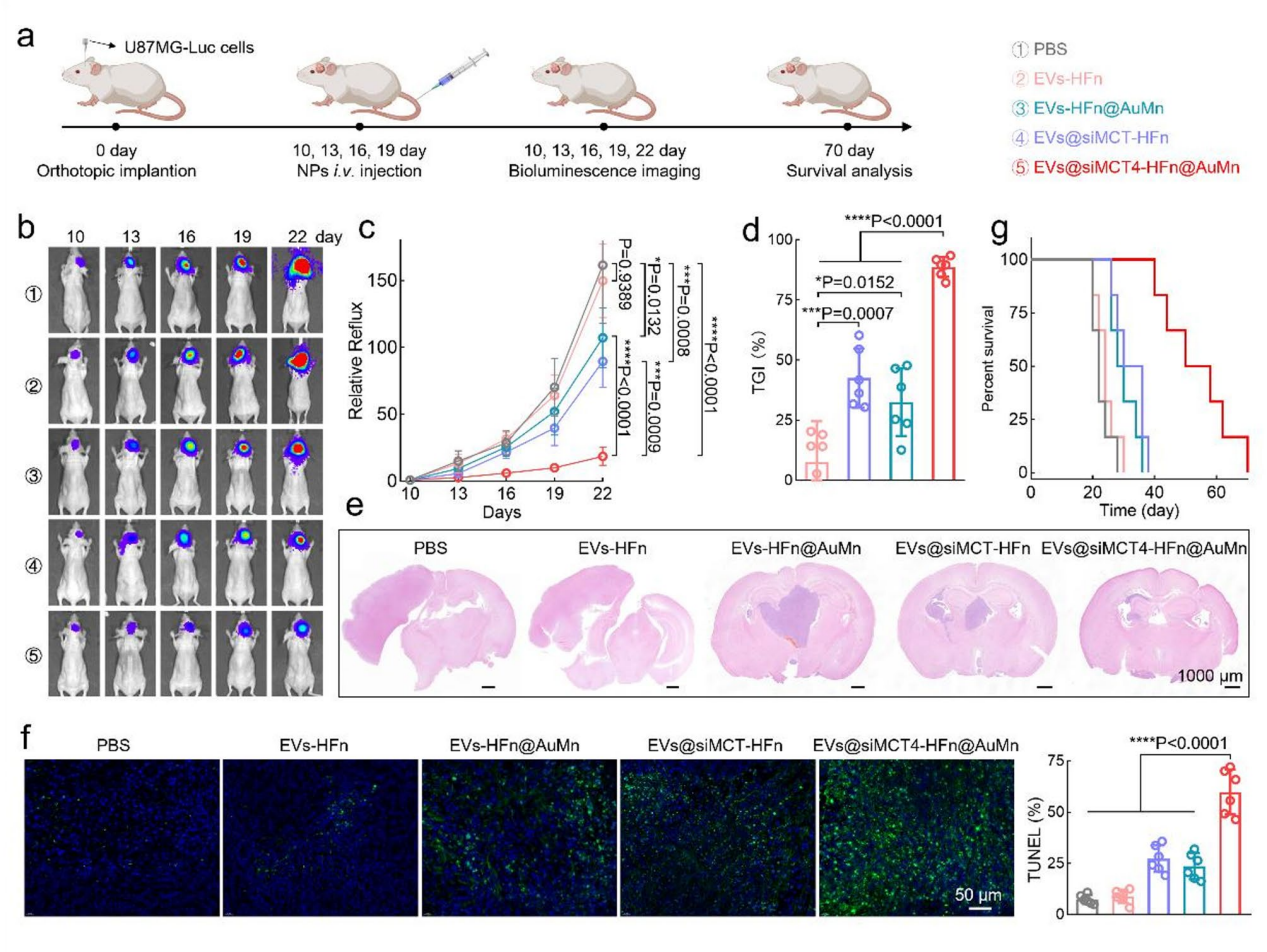
Evaluation of the therapeutic efficacy of EVs@siMCT4-HFn@AuMn in a U87MG-Luc glioma tumor model. (**a**) Experimental design for evaluating the efficiency of tumor inhibition after the indicated treatments, including PBS, EVs-HFn, EVs-HFn@MnAu, EVs@siMCT4-HFn, or EVs@siMCT4-HFn@AuMn. (**b**) Representative bioluminescence images of U87MG-Luc glioma-bearing mice receiving the indicated treatments at the indicated time points. (**c**) Quantification of the bioluminescence signal intensity of U87MG-Luc glioma cells from the indicated groups. (**d**) TGI rates of the different groups. The data were normalized to those of the PBS group. (**e**) Representative H&E staining images of brains obtained from mice receiving the indicated treatments. (**f**) TUNEL staining images of frozen tumor sections (left) and corresponding quantification analysis of TUNEL signals (right) in different groups. (**g**) Survival of mice receiving the indicated treatments. Data in c, d, and f are presented as the mean ± SD (*n* = 6). P values are calculated using one-way ANOVA

We next evaluated the in vivo therapeutic efficacy of various formulations. Orthotopic U87MG-Luc-bearing mice were randomly divided into five groups: PBS, EVs-HFn, EVs-HFn@MnAu, EVs@siMCT4-HFn, and EVs@siMCT4-HFn@AuMn. After intravenous injection of the various formulations, therapeutic efficacy was monitored *via* bioluminescence using the IVIS imaging system at the indicated time points (Fig. 5a). The results revealed that EVs@siMCT4-HFn@AuMn conferred the strongest antitumor activity, nearly completely suppressing GBM growth (Fig. 5b-c). Quantitative analysis on Day 22 revealed a tumor growth inhibition (TGI) rate of 88.7% for EVs@siMCT4-HFn@AuMn treatment (Fig. 5d), significantly outperforming EVs-HFn (7.7%), EVs-HFn@MnAu (32.5%), and EVs@siMCT4-HFn treatments (42.5%). Consistently, no significant body weight loss was observed in the mice treated with EVs@siMCT4-HFn (Fig. S10). Hematoxylin and eosin (H&E) staining of whole brains also confirmed that EVs@siMCT4-HFn@AuMn-treated mice presented the smallest tumor size compared with all other treatment groups (Fig. 5e). Additionally, TUNEL staining revealed extensive tumor apoptosis in the EVs@siMCT4-HFn@AuMn group (Fig. 5f). As a result, a markedly extended lifespan of the mice to a median survival time of 52 days was achieved with EVs@siMCT4-HFn@AuMn treatment (Fig. 5g).

The therapeutic synergy of EVs@siMCT4-HFn@AuMn was further validated in an orthotopic GL261 glioma model (Fig. S11a-c). Notably, the combination therapy with anti-PD-1 antibody (αPD-1) showed particularly remarkable efficacy, with 75% of the treated mice achieving long-term survival (> 100 days) without any tumor recurrence (Fig. S11d). Furthermore, the combination therapy significantly expanded effector memory T cells (T_EM_) (Fig. S12), suggesting the potential for durable anti-tumor immunity and protection against glioma rechallenge. Mechanistic investigations revealed that EVs@siMCT4-HFn@AuMn treatment effectively reprogrammed the immunosuppressive TME, characterized by enhanced infiltration of M1-type macrophages, mature DCs, and CD8^+^ T cells, along with decreased regulatory T cells accumulation (Fig. S13). Further transcriptomic profiling revealed that this treatment modulated critical cellular processes including metabolism, oxidative stress response, and immune activation pathways (Fig. S14). These results collectively highlight the multimodal therapeutic mechanism of our approach, which synergistically integrates ST, CDT, LA metabolic therapy, and immunomodulation for effective GBM treatment.

Finally, the potential toxicity of EVs@siMCT4-HFn@AuMn was evaluated. As showed in Fig. S15a-d, no abnormal biochemical markers, systemic cytokine (IL-6, TNF-α, and IFN-γ), or histological abnormalities were observed. Additionally, hemolysis experiments demonstrated that EVs@siMCT4-HFn@AuMn nanoparticles did not induce significant hemolytic effects (Fig. S15e), supporting the safety of EVs@siMCT4-HFn@AuMn treatment. Such a good safety should be primarily attributed to the remarkable targeting specificity of EVs@siMCT4-HFn@AuMn, which effectively minimizes off-target exposure of the therapeutic formulation to normal organs and healthy brain tissue. Collectively, our comprehensive results demonstrate that this engineered EV-HFn-based nanoplatform represents a highly promising therapeutic strategy for GBM treatment, combining superior antitumor efficacy with minimal systemic toxicity.

## Conclusion

In summary, we successfully developed EVs@siMCT4-HFn@AuMn, a dual targeting and synergistic therapeutic system for the effective treatment of GBM, a highly challenging malignancy. We demonstrated that the incorporation of HFn on M1EVs enhanced their BBB penetration and GBM tissue/cells-targeting abilities. By separately loading siMCT4 and ultrasmall Au-MnO_2_ NPs into the vesicles, we confirmed that developed EVs@siMCT4-HFn@AuMn exhibited the desired properties for synergistic therapy combining ST, CDT, LA metabolic therapy, and immunomodulation. The significant inhibition of tumor growth in the U87MG xenograft glioma model underscores the versatility of this nanoplatform, supporting innovative multimodal therapeutic strategies for GBM.

### Experimental section

#### Materials and reagents

Potassium permanganate was purchased from Beijing Chemical Works. Chloroauric acid trihydrate (HAuCl_4_) was obtained from MACKLIN (Shanghai, China). NHS-PEG_5_-DBCO and N_3_-Cy5.5 were sourced from Click Chemistry Tools. Methylene blue (MB), 2′,7′-dichlorofluorescin diacetate (DCFH-DA), glucose detection kit, lactate detection kit, Cell Counting Kit-8 (CCK-8), apoptosis analysis kit, and Hoechst 33,342 were procured from Solarbio^®^life sciences. 5,5-dimethyl-1-pyrroline N-oxide (DMPO) was purchased from Santa Cruz Biotechnology. 2-deoxy-2-[(7-nitro-2,1,3-benzoxadiazol-4-yl) amino]-D-glucose (2-NBDG) was obtained from Abnova. Anti-MCT4 antibody were purchased from Abcam. FITC-anti-CD86 and APC-anti-CD206 were obtained from BioLegend.

#### Cell lines

The glioblastoma cell lines (U87MG and U87MG-Luc, GL261-Luc) were obtained from the American Type Culture Collection (ATCC) and were maintained in our laboratory (Shenzhen Second People’s Hospital, Shenzhen, China). The human brain endothelial cell line (hCMEC/D3) was purchased from BeNa Culture Collection. Human brain vascular pericytes and astrocytes were purchased from Sciencell. The cells were cultured in Dulbecco’s modified Eagle’s medium (DMEM) supplemented with 10% fetal bovine serum (FBS, Gibco) and 1% (v/v) penicillin-streptomycin (Sigma-Aldrich).

#### Animals

Balb/c nude and C57BL/6 mice (6–8 weeks) were purchased from Beijing Vital River Laboratory. The animal protocol was approved by the ethics review committee of Longgang Central Hospital on Laboratory Animal Care (approval ID: NO.2024-093). All the mice were housed in IVC mouse cages and maintained in specific pathogen-free animal facilities under a 12-hour light/dark cycle at 23 °C with 55 ± 5% humidity.

### Preparation of EVs-HFn and EVs@siMCT4-HFn@AuMn

Preparation of EVs-HFn: N_3_-modified EVs (N_3_-EVs) were prepared according to our previously reported methods [31, 32]. Briefly, microglial HMC3 cells were subjected to 1 mg/mL lipopolysaccharide (LPS) and 0.8 mM azidoethyl-choline for 24 h to obtain N_3_-modified M1-type HMC3 cells. N_3_-EVs were isolated from the cell supernatant via ultracentrifugation. For the preparation of DBCO-modified HFn (DBCO-HFn), 2 µL of NHS-PEG_5_-DBCO (2 mM) was added to 100 µL of HFn solution (0.8 mg/mL) and incubated for 4 h at room temperature. The unreacted NHS-PEG_5_-DBCO was removed *via* ultrafiltration using an ultrafiltration device with a molecular weight cutoff of 10 kDa (Millipore). To prepare EVs-HFn, N_3_-EVs were mixed with DBCO-HFn at a 1:100 (particle number) ratio and incubated for 2 h at room temperature. The concentrations of EVs, N_3_-EVs, HFn, DBCO-HFn, and EVs-HFn were quantified using a BCA assay. The particle numbers of EVs and EVs-HFn were determined using nanoparticle tracking analysis (NTA).

Preparation of EVs@siMCT4: EVs and siMCT4 were mixed in a 4 mm cuvette (Bio-Rad, Hercules, CA, USA) at a 1:2 (w/w) ratio and pulsed at 100 V, 100 µF, and 200 Ω. After incubation at 37 °C for 30 min, the mixtures were ultrafiltered (8 500 g, 15 min) to remove the unloaded free siMCT4.

Preparation of HFn@AuMn: Ultrasmall nano-Au and MnO_2_ were prepared according to previously established protocols [33, 34] and subsequently loaded into HFn based on the disassembly and self-assembly capability of HFn. In detail, HFn (1 mg/mL) in urea (8 M) was vortexed at room temperature for 30 min to dissociate HFn into protein monomers. Ultrasmall nano-Au and MnO_2_ were then added to the solution at a final concentration of 1 mg/mL each. After vortexing for 15 min, the mixture was dialyzed at 4 °C in gradient concentrations of urea (7, 5, 3, 2, 1, and 0 M, each for 4 h) to slowly reassemble the HFn protein cages, yetlding HFn@AuMn. The contents of Au and Mn in HFn@AuMn were determined using inductively coupled plasma optical emission spectrometry (ICP-OES).

Preparation of EVs@siMCT4-HFn@AuMn: Similar to EVs-HFn, EVs@siMCT4-HFn@AuMn was prepared through click chemistry by mixing EVs@siMCT4 and HFn@AuMn.

### Characterizations of EVs-HFn and EVs@siMCT4-HFn@AuMn

For TEM imaging of N_3_-EVs, HFn, DBCO-HFn, and EVs-HFn, samples dispersed in PBS were dropped onto 300-mesh copper grids and negatively stained with a 2% phosphotungstic acid solution. Images were acquired using an HT7000 microscope (Hitachi, Japan) at a voltage of 120 kV. TEM images of MnO_2_, Au, and HFn@MnAu without negative staining were observed using an HT7000 microscope. The hydrodynamic diameter and zeta potential of various NPs were detected using a dynamic light scattering system (NANO ZS, Malvern).

To assess siMCT4 loading, 100 µL (1 mg/mL) of EVs were incubated with 1 µL of DiO for 30 min at 37 °C to label EVs with DiO. The unbound DiO was removed using a NAP-5 column, following the manufacturer’s instructions. The DiO-labeled EVs were then mixed with Cy5-labeled siMCT4. After electroporation, the resulting siMCT4 (Cy5)-loaded EVs (DiO) were observed using confocal laser scanning microscopy (CLSM, UltraVIEW VoX, PerkinElmer, USA).

To evaluate DBCO modification, HFn and DBCO-HFn (20 µg/mL) were reacted with N_3_-Cy5.5 for 2 h at room temperature. After the removal of unbound N_3_-Cy5.5 *via* ultrafiltration, the fluorescence spectra of the samples were measured using a Fluoromax-4 spectrofluorometer (HORIBA JOBIN YVON).

For glucose consumption activity assessment, EVs@siMCT4-HFn (lacking ultrasmall nano-Au and MnO_2_) and EVs@siMCT4-HFn@AuMn (at an equal concentration of protein: 100 µg/mL) were added to a 1 mM glucose solution and incubated for different durations. The concentrations of glucose and gluconic acid were determined using a glucose detection kit.

To evaluate hydroxyl radicals·(•OH) production, EVs@siMCT4-HFn@AuMn (100 µg/mL) was incubated with MB (10 µg/mL) in the presence or absence of 1 M glucose. The absorbance of MB at 664 nm was measured at the indicated time points using an automatic microplate reader. The production of •OH by EVs@siMCT4-HFn@AuMn was also detected using an ESR spectrometer (Bruker E500 spectrometer) following the addition of an •OH probe (100 µM DMPO).

### In vitro antitumor efficacy evaluation

To evaluate the glucose consumption of various NPs in vitro, U87MG cells were seeded in 24-well plates or confocal dishes (1 × 10^5^ cells per well) and incubated overnight at 37 °C. EVs-HFn, EVs@siMCT4-HFn, EVs-HFn@AuMn, or EVs@siMCT4-HFn@AuMn (at an equal concentration of protein: 200 µg/mL) resuspended in fresh cell culture medium were added to the cells and incubated for 12 h. Following the manufacturer’s instructions, a glucose uptake and transport assay kit (2-NBDG) was added, and glucose levels in the treated cells were imaged via confocal laser scanning microscopy (CLSM) or analyzed using flow cytometry.

To assess ROS generation in vitro, cultured U87MG cells were treated with various NPs as described above. After incubation with an ROS probe (DCFH-DA) for 30 min, the cells were imaged using CLSM.

To evaluate MCT4 expression in cells, cultured U87MG cells were treated with various NPs as described above. After 24 h of incubation, all treated cells were fixed with 4% paraformaldehyde (15 min), permeabilized with 0.25% Triton X-100 (10 min), blocked with 1% (w/v) BSA (30 min), and incubated overnight with an anti-MCT4 antibody. The cells were then stained with fluorescent secondary antibodies (30 min) and observed using CLSM after the cell nuclei were labeled with Hoechst 33,342.

In vitro cytotoxicity was determined using a CCK-8 assay. U87MG cells were seeded in 96-well plates (1 × 10^4^ cells per well) and incubated overnight at 37 °C. After treatment with different concentration of EVs-HFn, EVs@siMCT4-HFn, EVs-HFn@AuMn, or EVs@siMCT4-HFn@AuMn for 24 h, the CCK-8 kit reagent was added, and the cells were incubated for an additional 2 h. Cell viability was evaluated using an automatic microplate reader. For the apoptosis assays, the cells that received the indicated treatments were analyzed by flow cytometry after they were stained with Annexin V-FITC/PI according to the manufacturer’s instructions.

### In vitro evaluation of subcellular localization

hCMEC/D3 cells (1 × 10^5^) were seeded in confocal dishes and incubated overnight. FITC labeled-HFn, EVs-HFn, or anti-TIM-2 antibody (anti-HFR antibody, HFR Ab) resuspended in fresh cell culture medium were added to the cells and incubated for 2 h. Following three PBS washes, cells were fixed with 4% formaldehyde, permeabilized with 0.1% Triton X-100, and blocked with 3% normal goat serum. For subcellular localization analysis, cells were subsequently incubated with AlexaFluor555-conjugated anti-Lamp1 monoclonal antibody (for lysosomal visualization) and secondary antibodies for HFR Ab detection at 37 °C for 1 h. Fluorescence imaging was performed using CLSM.

### BBB penetration and tumor-targeting ability evaluation

To assess the BBB penetration and tumor cell-targeting abilities of EVs-HFn in vitro, a classical BBB model was established following our previous methods [6, 36]. hCMEC/D3 cells (1 × 10^4^) and a co-culture of human pericytes and astrocytes were separately plated on the top and reverse side of a 0.4-µm pore size transwell membrane of the transwell™ coculture system. U87MG cells (2 × 10^4^) were seeded in the lower chamber. Once the transepithelial electrical resistance (TEER) of the model reached 150 Ω cm^2^, EVs(Cy5), EVs(Cy5)-HFn, or EVs@siMCT4-HFn(FITC)@AuMn (100 µg protein/mL) was added to the upper chamber. To visualize the uptake of EVs or EVs-HFn by U87MG and hCMEC/D3 cells, the vesicle-treated cells were fixed with 4% paraformaldehyde and labeled with phalloidine (for hCMEC/D3 cells and BBB layer) or Hoechst 33,342 (for U87MG cells), followed by imaging using CLSM (NIKON). To assess the time-dependent uptake behavior of Cy5-labeled EVs or EVs-HFn, U87MG cells were collected at different time points and analyzed using flow cytometry (Beckman Coulter, CytoFLEX). To verify the BBB penetration *via* TEM, the sample from the lower chamber of the in vitro BBB model treated with EVs@siMCT4-HFn@AuMn was negatively stained with a 2% phosphotungstic acid and subsequently examined using an HT7000 microscope.

For in vivo assessment of tumor-targeting performance, 3 µL of U87MG-Luc cells (3 × 10^5^) were stereotactically injected into the brain parenchyma of Balb/c nude mice. The successful establishment of the U87MG-Luc-bearing mouse model was confirmed by bioluminescence using an IVIS imaging system. Following this, the glioma-bearing mice were randomly divided into two groups and intravenously injected with DIR-labeled EVs, EVs-HFn, or EVs@siMCT4-HFn@AuMn (200 µg protein per mouse). Fluorescence images of the mice were taken at the indicated time points (0, 1, 4, 12, 24, 48, and 72 h). The excised major organs (heart, liver, spleen, lung, and kindey) and brains obtained from the mice at 24 h postinjection were also visualized using the IVIS imaging system. For section imaging, U87MG-Luc-bearing mice were injected with DiO-labeled EVs or EVs-HFn. The dissected major organs and brains were fixed with 4% paraformaldehyde, dehydrated with 30% sucrose solution, embedded in optimal cutting temperature (OCT) tissue compound, and finally sectioned into 8 μm slides. After labeling with DAPI, the slides were imaged using CLSM.

### Evaluations of antitumor effect against CDX model

U87MG-Luc glioma-bearing Balb/c nude mice were randomly divided into five groups (six mice per group) after confirming tumor growth via bioluminescence using the IVIS imaging system on day 10. PBS, EVs-HFn, EVs@siMCT4-HFn, EVs-HFn@AuMn, EVs@siMCT4-HFn@AuMn (200 µg protein per mouse) were intravenously injected into the mice on days 10, 13, 16, and 19. Throughout the experiment, glioma growth was monitored *via* bioluminescence using the IVIS imaging system. Body weight and survival of the mice were recorded. In parallel, three Balb/c nude mice from each group were randomly sacrificed 24 h after intravenous injection of the various formulations, and their brains were collected for TUNEL staining analysis. The remaining mice in each group were sacrificed on day 20 to obtain their brains for H&E staining analysis. The collected brains successively performed fixation (4% paraformaldehyde), dehydration (30% sucrose solution), embedding (OCT tissue compound), and sectioning. The slides were then stained with H&E or TUNEL kit according to the manufacturer’s instructions.

To explore the therapeutic efficacy of EVs@siMCT4-HFn@AuMn combined with αPD-1, hTFR1 expressed-GL261-Luc orthotopic glioma-bearing C57BL/6 mice were divided into seven groups (six mice per group) and intravenously injected with PBS, EVs-HFn, EVs@siMCT4-HFn, EVs-HFn@AuMn, EVs@siMCT4-HFn@AuMn (200 µg protein per mouse), αPD-1 (100 µg per mouse), or EVs@siMCT4-HFn@AuMn + αPD-1. NPs and αPD-1 were administered *via* intravenous and intraperitoneal injection, respectively. Tumor growth was monitored *via* bioluminescence using the IVIS imaging system.

For immune cell analysis, brain tumors were collected from mice one day after the final administration in the three-dose regimen. After homogenization into single-cell mixtures. the cells were washed with PBS and stained with the addition of fluorescently labeled antibodies. For TAMs analysis, the cells were stained using Percp-CD11c, PB-F4/80, PE-CD86, and APC-CD206. For DC maturation analysis, the cells were stained using Percp-CD11c, FITC-CD80, and APC-CD86. For CD8^+^ T cells, CD4^+^ T cells, and Tregs analysis, the cells were stained with FITC-CD3, APC-Cy7-CD8, APC-CD4, PerCP-CD25, and PE-Foxp3. For T_EM_ analysis, the cells were stained with FITC-CD3, PerCP-CD8, PE-CD44, and APC-CD62L. After washing, the cells were detected *via* flow cytometry.

For RNA sequencing analysis, brain tumors were collected after treated with PBS or EVs@siMCT4-HFn@AuMn. Total RNA was subsequently extracted using TRIzol reagent following the manufacturer’s protocol, with RNA quality assessed using an Agilent 2100 Bioanalyzer. The obtained mRNA was enriched using Oligo (dT) magnetic beads and fragmented into short fragments using the fragmentation buffer. The resulting RNA fragments were then reverse transcribed into cDNA using primers. After purification with a QiaQuick PCR Extraction kit, the cDNA was sequenced on an Illumina HiSeq 2500 platform. Differential gene expression analysis was performed using the R package DEGseq, with significant differences defined by fold-change thresholds after appropriate normalization.

### Biosafety evaluation

Balb/c nude or C57BL/6 mice were intravenously injected with PBS or EVs@siMCT4-HFn@AuMn (200 µg protein per mouse) every 4 days for three times. Blood of C57BL/6 was collected at the indicated time points (6 h, 12 h, 24 h, day 3, day 7, and day 14) and centrifuged to collect serum (4000 rpm, 4 °C, 10 min) for ELISA analysis of IL-6, TNF-*α*, and IFN-*γ*. On day 14, serums and major organs (heart, liver, spleen, lung, kindeys) were collected. The serum levels of alanine aminotransferase (ALT), alkaline phosphatase (ALP), aspartate transaminase (AST), blood urea nitrogen (BUN), and lactate dehydrogenase (LDH) were measured using a biochemical autoanalyzer (TBA-40, Toshiba). The major organs (heart, liver, spleen, lung, kindey) and brains were stained with an H&E kit and imaged using an automatic multispectral imaging system (Vectra II, PerkinElmer). For the hemolysis experiments, saline solutions with different concentrations of EVs@siMCT4-HFn@AuMn nanoparticles were incubated with red blood cells at 37 °C for 2 h. After centrifugation, photographs of the supernatant were taken to assess color changes. The absorbance of collected supernatant at 541 nm was measured to determine the hemolysis rate. Red blood cells suspended in water and normal saline served as the positive and negative control group, respectively.

### Statistical analysis

All data were presented as mean ± SD. Statistical analysis was performed with GraphPad Prism 8.0.1 software by two-tailed unpaired Student’s *t*-tests, log-rank test, or one-way ANOVA.

## Supplementary Information

Supplementary Material 1

## Data Availability

No datasets were generated or analysed during the current study.
