# Supplementary material for: Ferritin-armed extracellular vesicles with enhanced BBB penetration and tumor-targeting ability for synergistic therapy against glioblastoma
Source: J Nanobiotechnology. 2025 Aug 18;23:570. doi: 10.1186/s12951-025-03646-x (PMC12360009; doi:10.1186/s12951-025-03646-x)
Supplement: Supplementary file 1 — Supplementary Material 1 [file 12951_2025_3646_MOESM1_ESM.docx]

Supporting Information

**Ferritin-armed extracellular vesicles with enhanced BBB penetration and tumor-targeting ability for synergistic therapy against glioblastoma**

Guihong Lu^1,2^, Peiling Zhuang^1^, Feng Li^3^, Fan Zhang^3^, Xiaoyan Li^1^, Weixiu Wang^1^, Hui Tan^1,2,*^

^1^ Institute of Pediatrics, Shenzhen Children's Hospital, Shenzhen, 518038, P. R. China.

^2^ Department of Neurosurgery, Health Science Center, The First Affiliated Hospital of Shenzhen University, Shenzhen Second People's Hospital, Shenzhen, 518035, P. R. China.

^3^ State Key Laboratory of Biopharmaceutical Preparation and Delivery, Institute of Process Engineering, Chinese Academy of Sciences, Beijing 100190, P. R. China.

* Correspondence E-mail: huitan@email.szu.edu.cn (H. Tan)


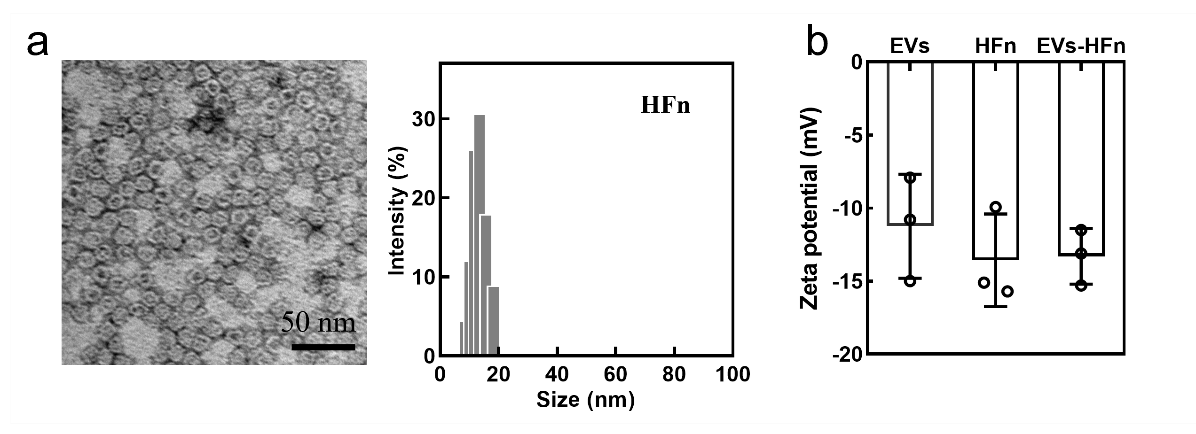


**Fig. S1 Construction of HFn and EVs-HFn.** (a) Representative TEM image and hydrodynamic diameter distribution of HFn. (b) Zeta potential of EVs, HFn, and EVs-HFn analyzed using DLS. Data in b are presented as the mean ± SD (n = 3).


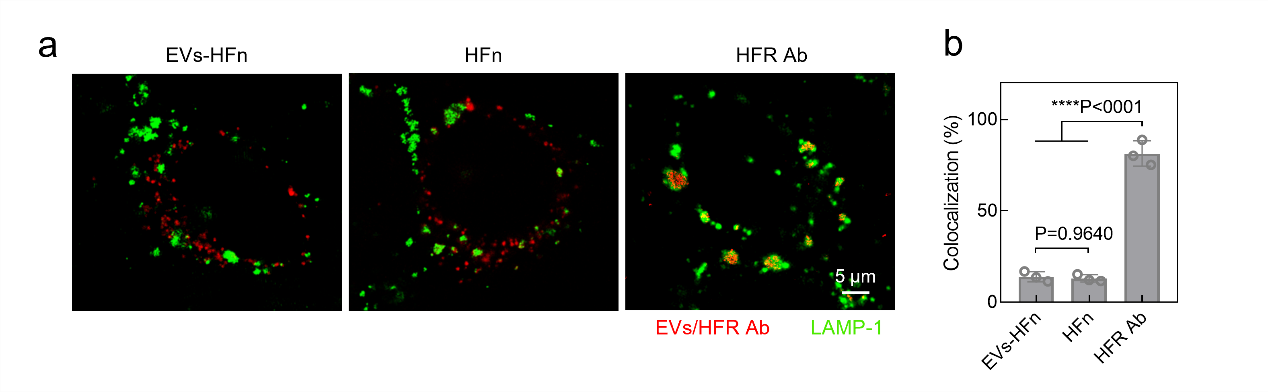


**Fig. S2 Subcellular localization of EVs-HFn, HFn, and anti-HFR antibody (HFR Ab) in hCMEC/D3 cells.** (a) CLSM images showing the lysosomal colocalization in hCMEC/D3 cells receiving the indicated treatment. (b) Percentage of lysosomal colocalization. Data in b are presented as the mean ± SD (n = 3). P values are calculated using one-way ANOVA.

**
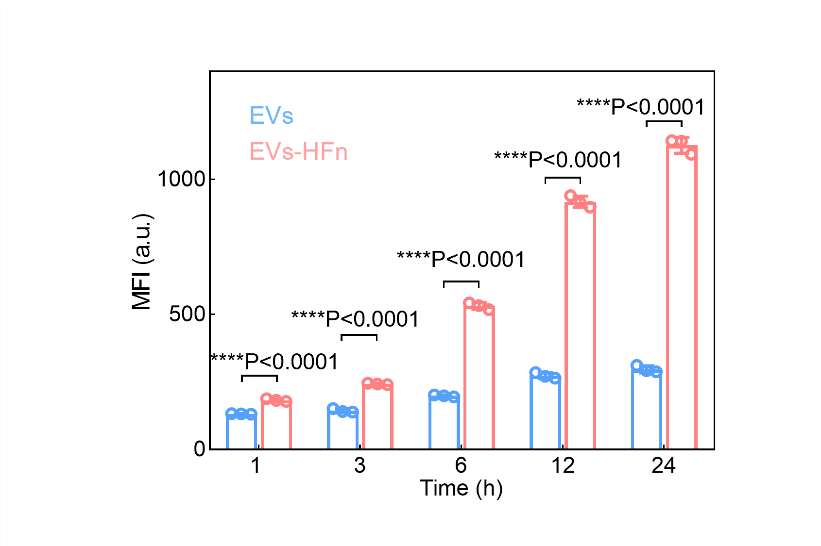
**

**Fig. S3** **Quantification analysis of time-dependent internalization of EVs or EVs-HFn by U87MG cells**. Data are presented as the mean ± SD (n = 3). P values are calculated using two-tailed unpaired Student’s *t*-test.


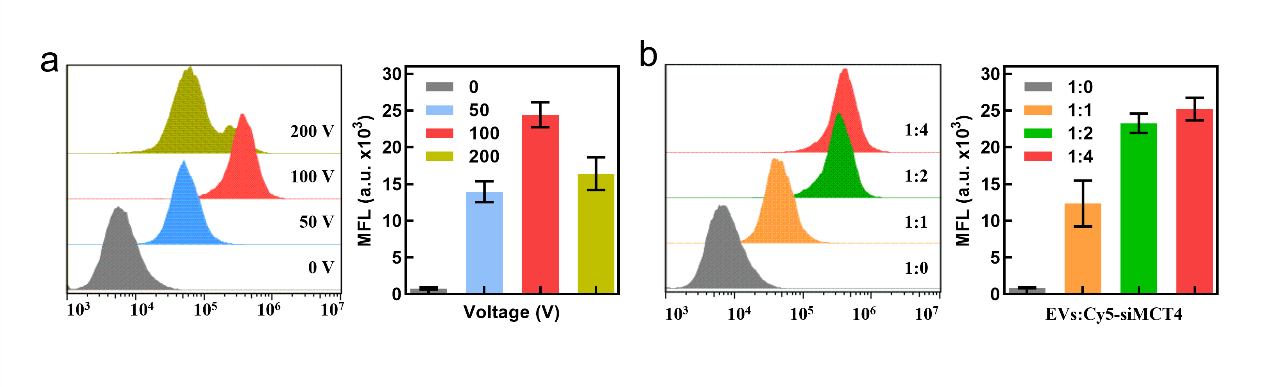


**Fig. S4 Condition optimization of siMCT4 electroporation into EVs.** (a) Flow cytometry analysis and the mean fluorescence intensities (MFI) of EVs after loading Cy5-siMCT4 by electroporation at 0 V, 50 V, 100 V, and 200 V, respectively. The MFI initially increased from 0 V to 100 V, and then reduced potentially due to electric pulse-triggered EVs broken. (b) Flow cytometry analysis and MFI of EVs@Cy5-with the ratios of EVs to Cy5-siMCT4 (w/w) were 1:0, 1:1, 1:2, and 1:4, respectively. The MFI increased from 1:0 to 1:2, then reached saturation at the ratios of 1:2 to 1:4. Data are presented as the mean ± SD (n = 3).


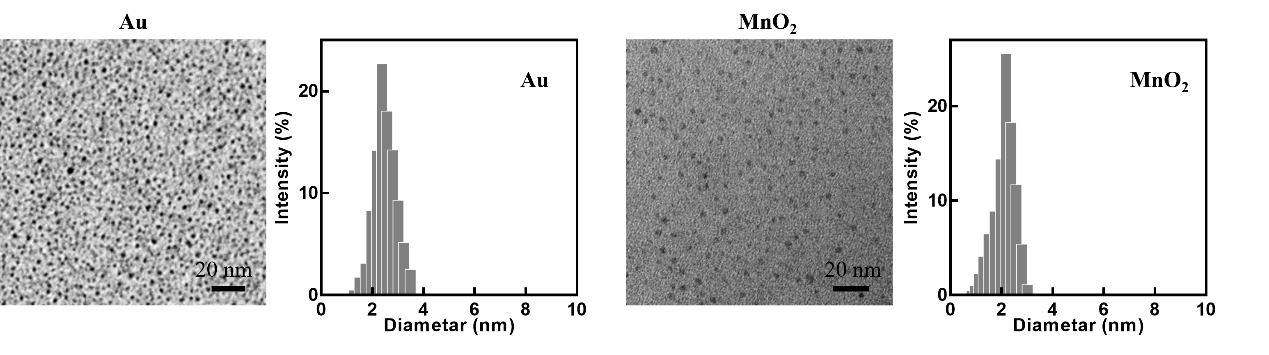


**Fig. S5** **Representative TEM images and hydrodynamic diameter distribution of ultrasmall nano-Au and MnO_2_**.

**
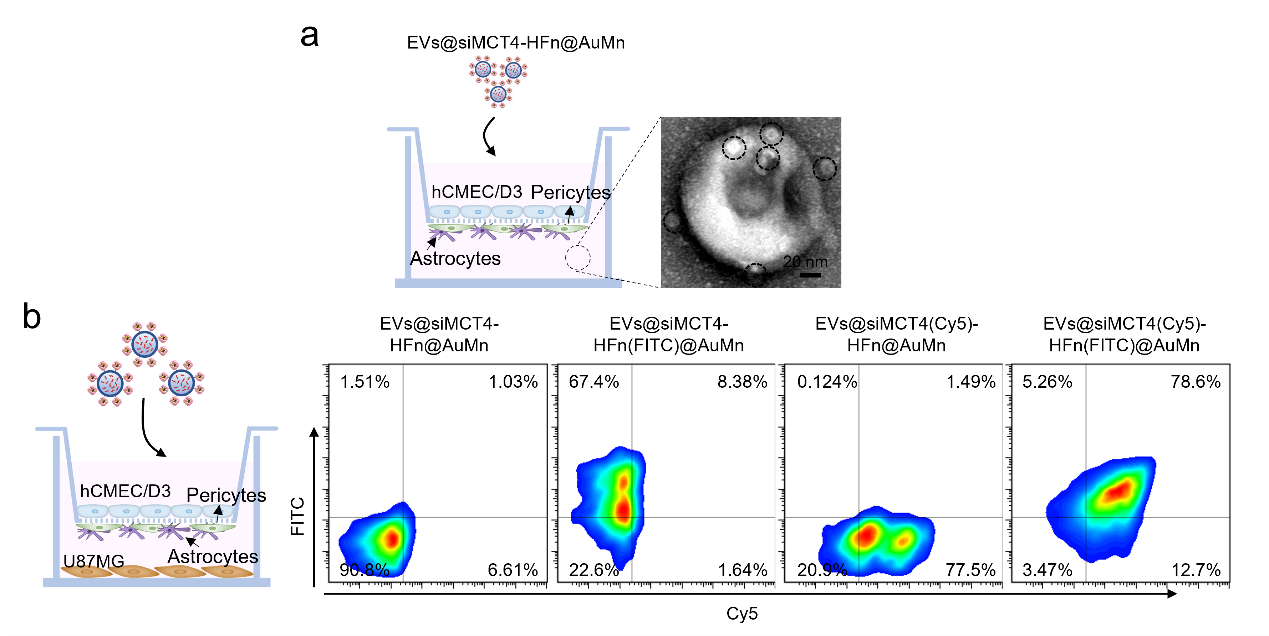
**

**Fig. S6** ***In vitro* BBB penetration of EVs@siMCT4-HFn@AuMn.** (a) Representative TEM of EVs@siMCT4-HFn@AuMn from the lower chamber (right). (b) Flow cytometry analysis of the U87MG cells showed the uptake of EVs@siMCT4-HFn@AuMn with the indicated fluorescence labeled.

**
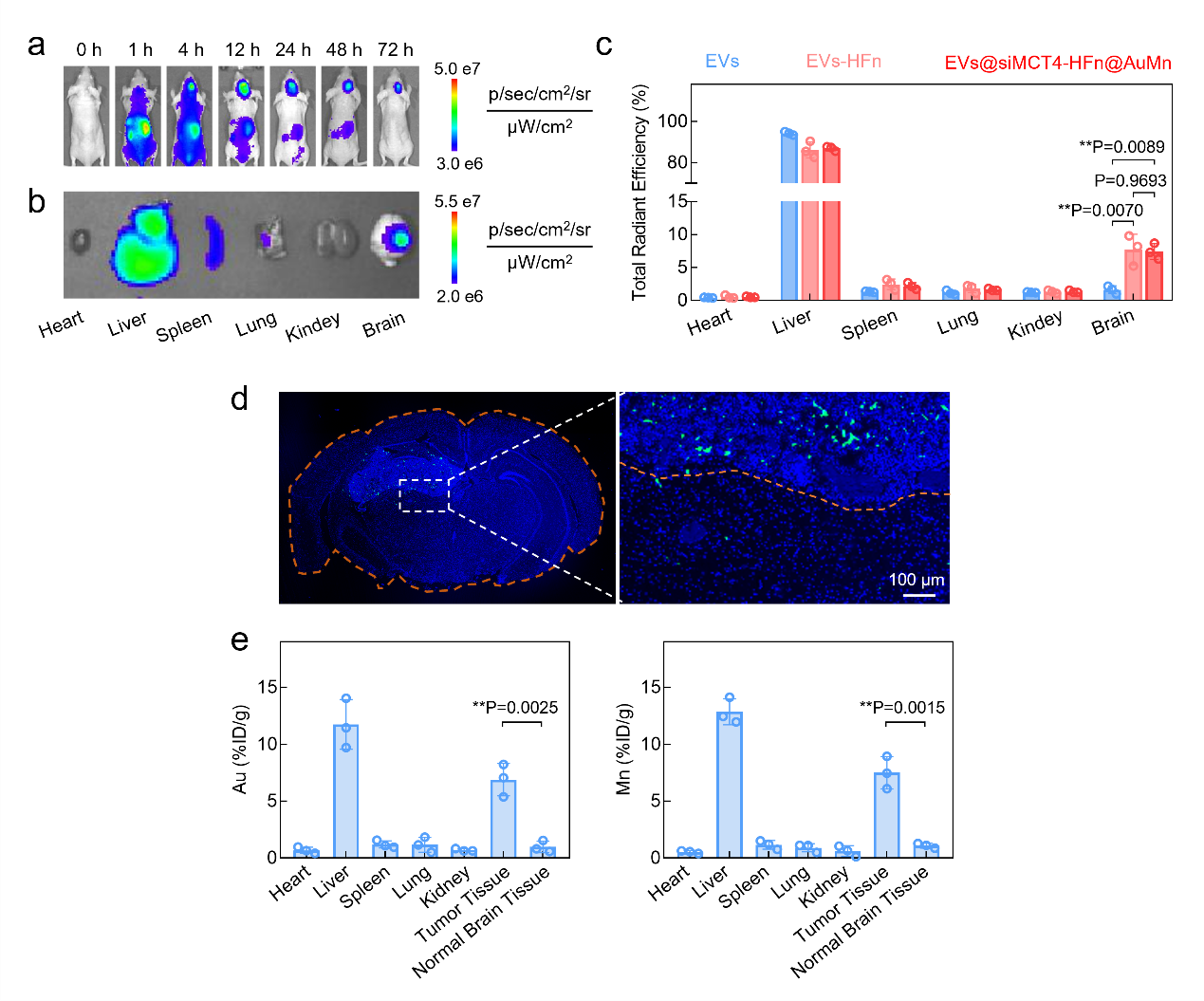
**

**Fig. S7** **Evaluation of the glioma targeting ability of EVs@siMCT4-HFn@MnAu in an orthotopic U87MG-luc glioma model.** (a) Real-time fluorescence images of glioma-bearing mice after *i.v.* injection with DIR-labeled EVs@siMCT4-HFn@MnAu. (b) Fluorescence image of excised main organs and brain at 24 h after administration. (c) The percentage of fluorescent signal in each organ in c and Fig. 2c. (d) Representative fluorescence image of frozen brain section showed the effective accumulation of EVs@siMCT4(Cy5)-HFn@MnAu. Blue: Hoechst 33342-labeled nuclei; green: Cy5-siMCT4. (e) Quantification of Au and Mn accumulation in different organs and tumors dissected from glioma-bearing mice 12 h after *i.v.* injection of EVs@siMCT4-HFn@MnAu. Data in c and e are presented as the mean ± SD (n = 3). P values are calculated using one-way ANOVA (b) or two-tailed unpaired Student’s *t*-test (e).


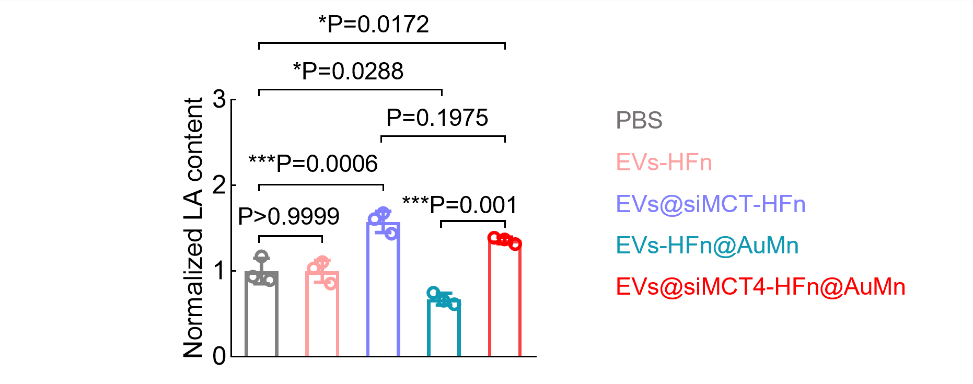


**Fig. S8 Normalized intracellular LA content after the indicated treatments.** Data are presented as the mean ± SD (n = 3). P values are calculated by using one-way ANOVA.


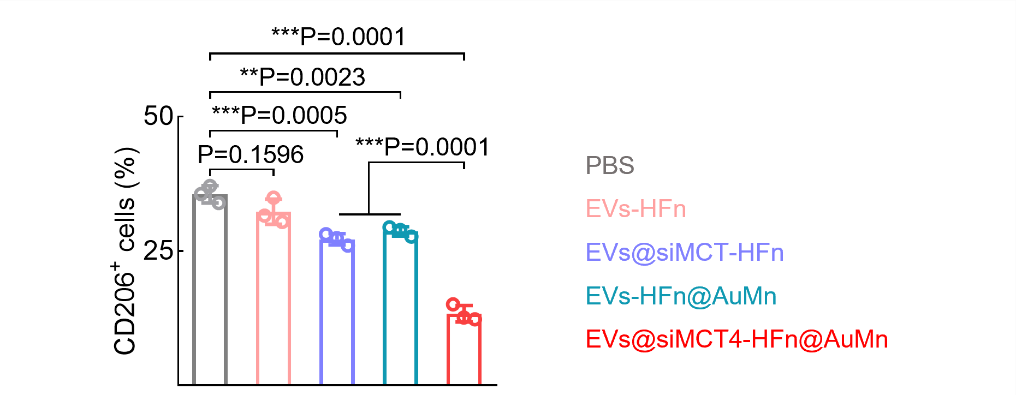


**Fig. S9 CD206-positive cells after the indicated treatments.** Data are presented as the mean ± SD (n = 3). P values are calculated by using one-way ANOVA.


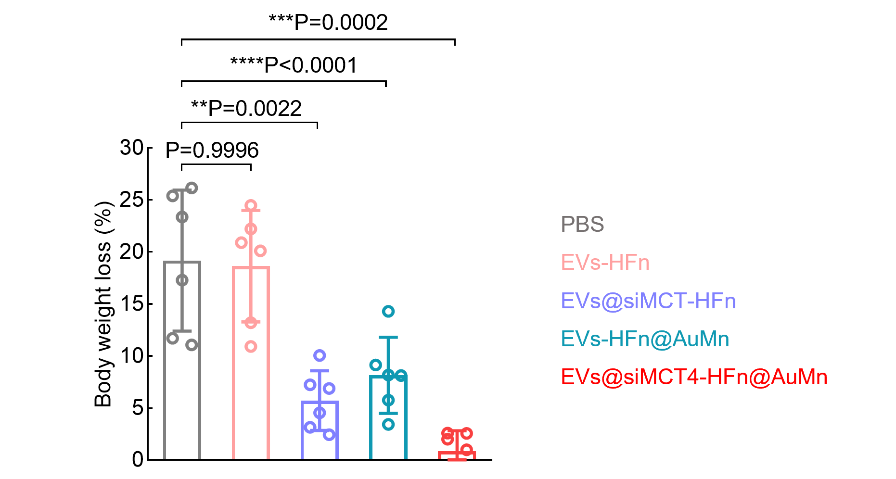


**Fig. S10 Body weight loss of mice receiving the indicated treatments (n = 3).** Data are presented as the mean ± SD. P values are calculated by using one-way ANOVA.


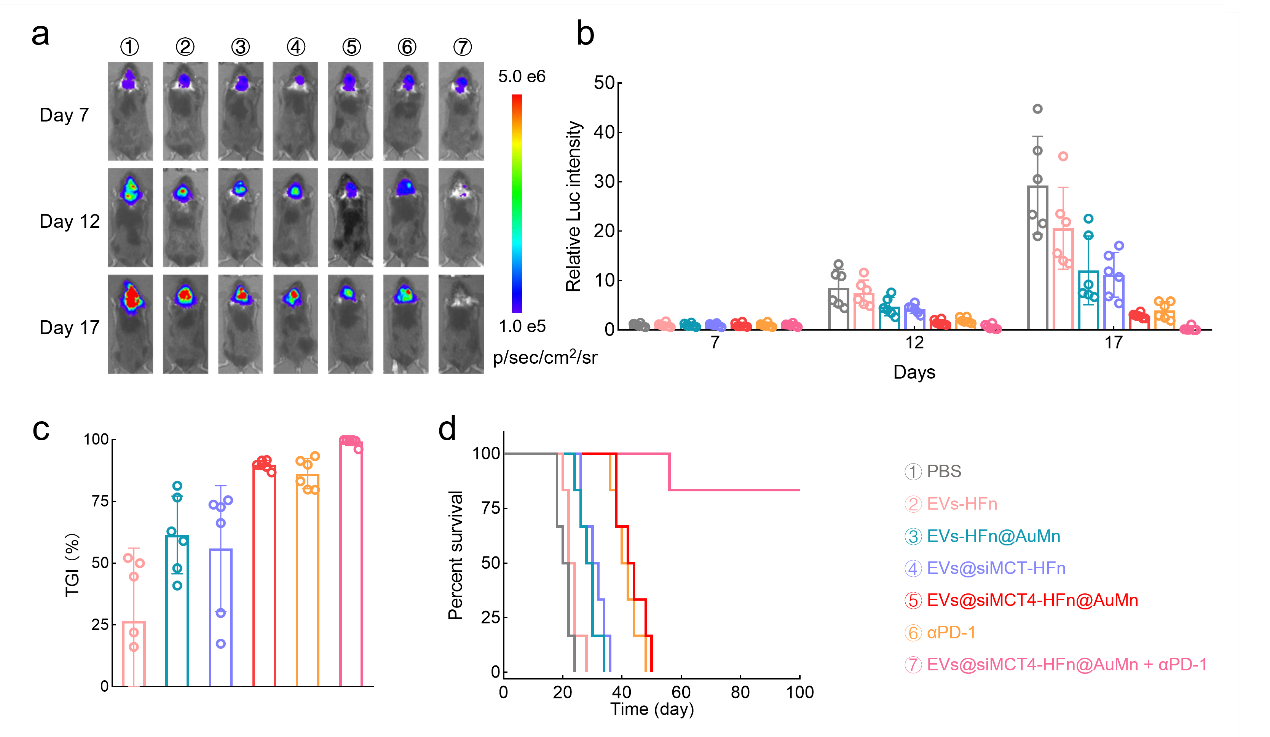


**Fig. S11 Evaluation of the therapeutic efficacy of EVs@siMCT4-HFn@AuMn in a GL261-Luc glioma tumor model.** (a) Representative bioluminescence images of GL261-Luc glioma-bearing mice receiving the indicated treatments. (b) Quantification of the bioluminescence signal intensity of GL261-Luc glioma from indicated groups. (c) TGI rate in different groups. The data were normalized to those of the PBS group. (d) Survival of mice receiving the indicated treatments. Data in b and c are presented as the mean ± SD (n = 6).


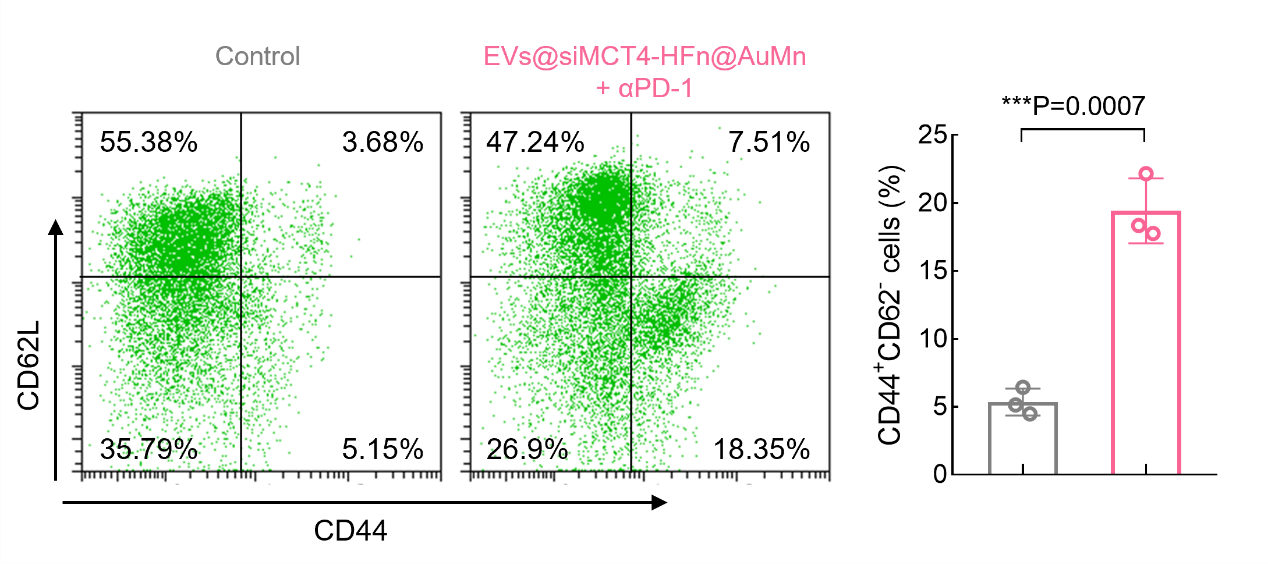


**Fig. S12 Representative flow cytometry plots and the corresponding quantitative analysis of T_EM_ cells (gated on CD3^+^CD8^+^CD44^+^CD62L^-^) in the lymph nodes of untreated control and EVs@siMCT4-HFn@AuMn + αPD-1 treated mice.** Data are presented as the mean ± SD (n = 3). P values are calculated using two-tailed unpaired Student’s *t*-test.


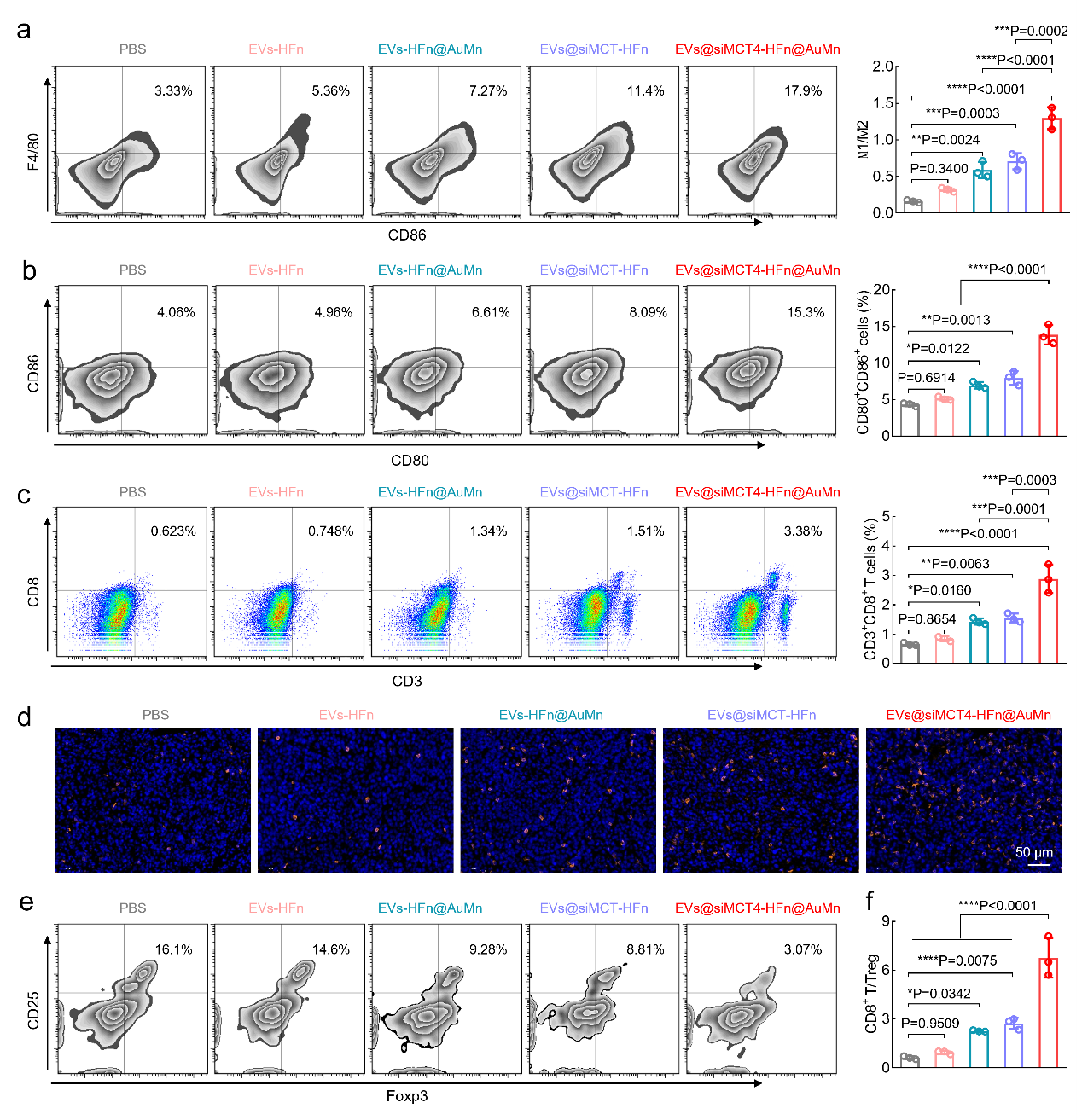


**Fig. 13** **Immunomodulatory Evaluation of EVs@siMCT4-HFn@AuMn.** (a) Representative flow cytometry plots of M1-like macrophages (gated on CD11b^+^F4/80^+^CD86^+^) and the M1/M2 ratio in tumors. (b) Representative flow cytometry plots and the corresponding quantitative analysis of mature DCs (gated on CD11c^+^CD80^+^CD86^+^). (c) Representative flow cytometry plots and corresponding quantitative analysis of CD8^+^ T cells. (d) Immunofluorescence staining analysis of CD8^+^ T cell in tumor tissues. Blue: nuclei. (e) Representative flow cytometry plots of Tregs (gated on CD4^+^CD25^+^Foxp3^+^). (f) The CD8 T cell/Treg ratio in tumors. Data in a-c and f are presented as the mean ± SD (n = 3). P values are calculated using one-way ANOVA.


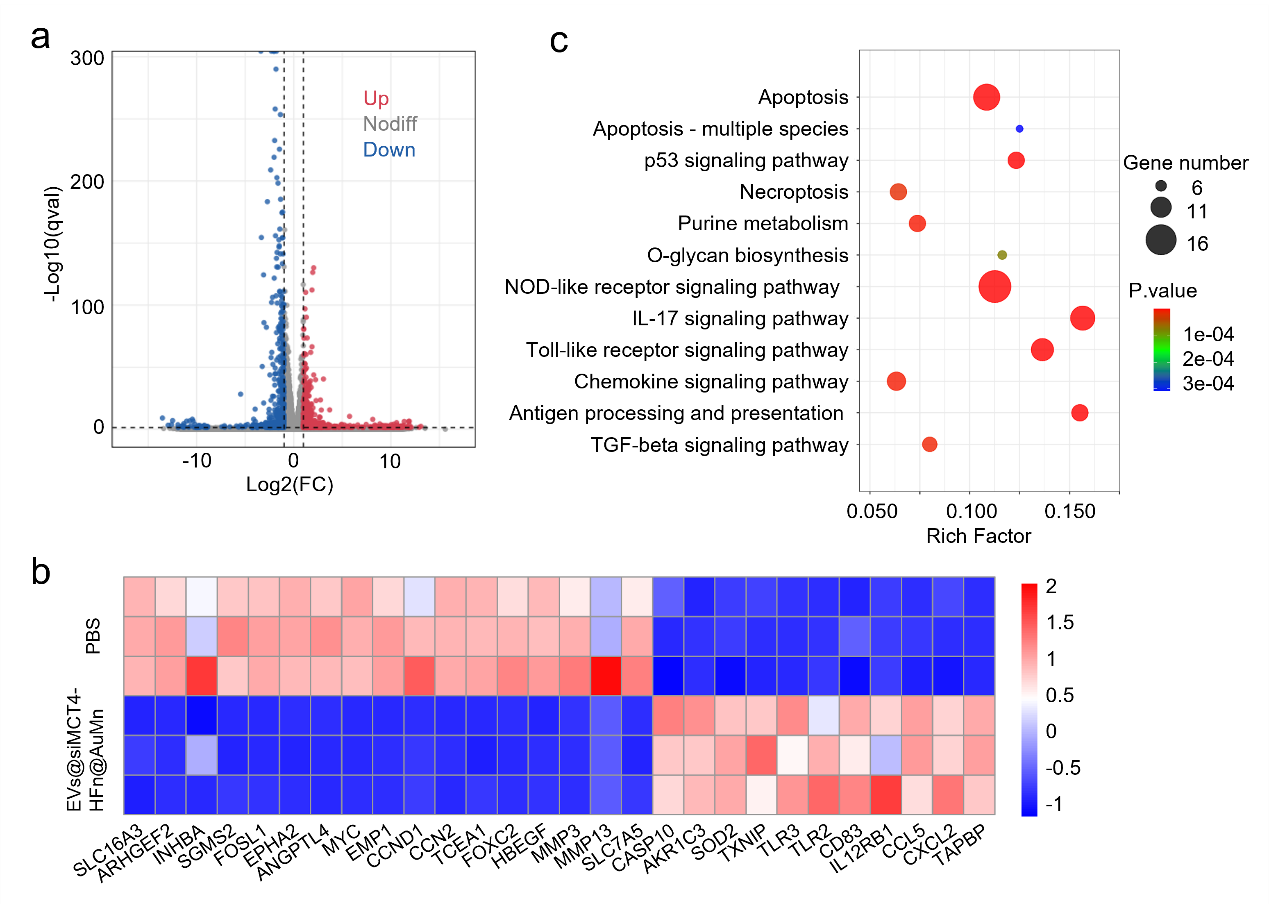


**Fig. S14** Transcriptome analysis of tumors after treatment with PBS or EVs@siMCT4-HFn@AuMn. (a) Volcano plots of upregulated and downregulated genes between PBS and EVs@siMCT4-HFn@AuMn groups (fold change≥2 and *q <* 0.05). (b) Heatmap of differentially expressed genes involved in metabolism, oxidative stress, immune, and cell apoptosis. (c) Kyoto Encyclopedia of Genes and Genomes (KEGG) enrichment analysis of the differentially expressed genes between PBS and EVs@siMCT4-HFn@AuMn groups.

Volcano plots identified 1187 significant differentially expressed genes (DEGs), comprising 492 downregulated and 695 upregulated genes (a). Notably, tumors treated with EVs@siMCT4-HFn@AuMn showed increased expression of oxidative stress-related (SOD2, TXNIP), apoptosis-related (CASP10, AKR1C3), and immune activation-related genes (TLR3, CD83, CCL5), while exhibiting decreased expression of metabolic (SLC16A3, SLC7A5), oncogenic (MYC, CCND1, CCN2), and immunosuppressive genes (b). KEGG pathway analysis further demonstrated that the treatment significantly affected pathways related to cell proliferation/apoptosis (p53 signaling pathway), metabolism (purine metabolism), and immune regulation (Toll-like receptor signaling pathway, TGF-beta signaling pathway, chemokine signaling pathway) (c).


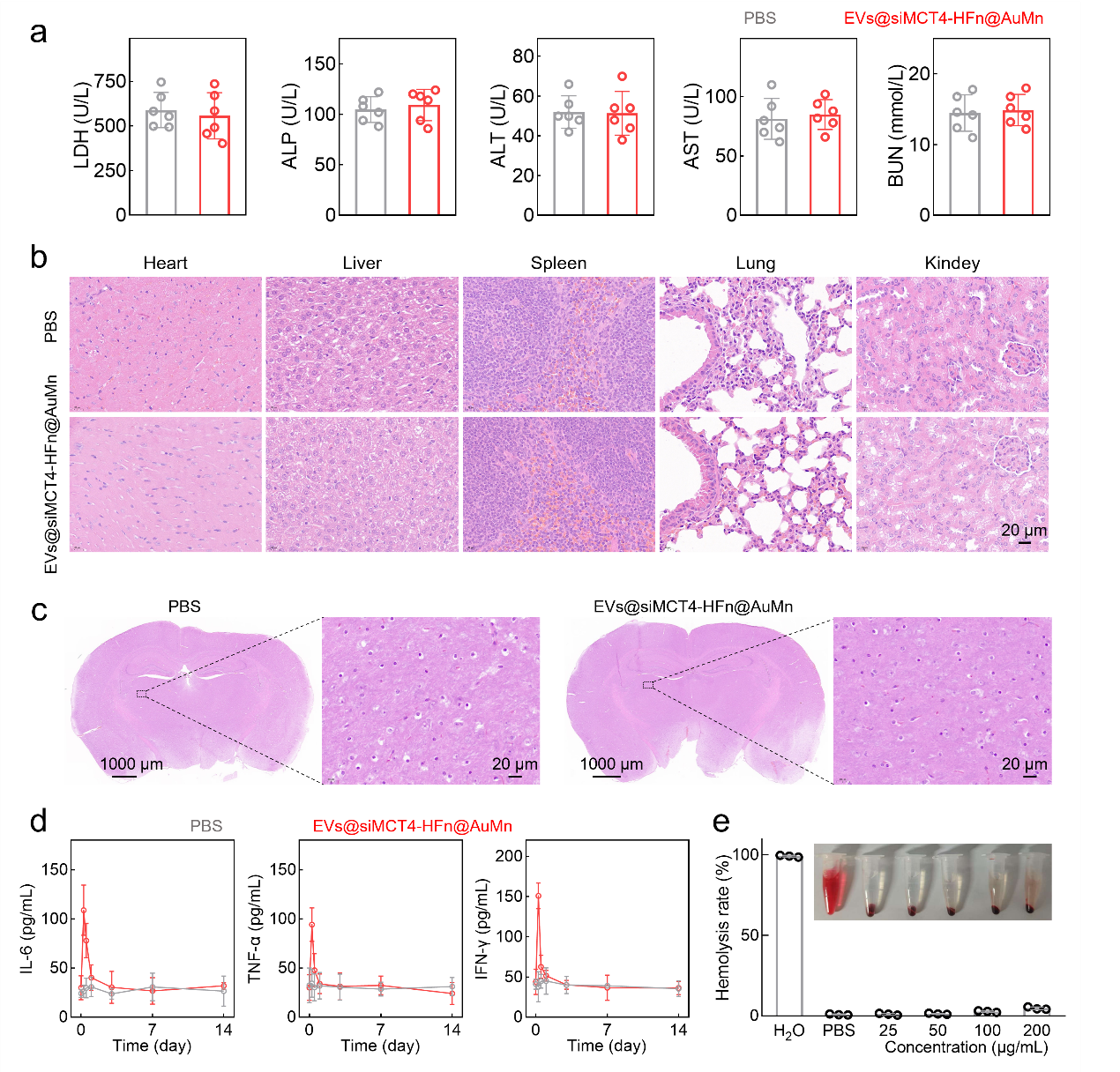


**Fig. S15 Bio-safety evaluation of EVs@siMCT4-HFn@AuMn.** (a) Hematological parameter detection in Balb/c nude mice receiving PBS or EVs@siMCT4-HFn@AuMn treatment (n = 6)**.** The blood urea nitrogen (BUN), aspartate alanine aminotransferase (ALT), aminotransferase (AST), alkaline phosphatase (ALP), and lactate dehydrogenase (LDH) levels were all within normal range. (b) H&E staining of heart, liver, spleen, lung, and kidney slices. No abnormality was found in these major organs obtained from mice receiving EVs@siMCT4-HFn@AuMn treatment. (c) H&E staining of brains obtained from mice receiving PBS or EVs@siMCT4-HFn@AuMn treatment. (d) Systemic cytokines (IL-6, TNF-α, and IFN-γ) release profiles at different time points (n = 3). (e) The hemolysis rate of red blood cells following incubation with varying concentrations of EVs@siMCT4-HFn@AuMn (n = 3). Data in a, d, and e presented as the mean ± SD.
